# Disparity of metatibial and metatarsal cuticular and sensory structures in Cixiidae (Hemiptera: Fulgoromorpha) with a metatibiotarsal diagnosis for the tribes

**DOI:** 10.1186/s40851-024-00239-8

**Published:** 2024-08-26

**Authors:** Jolanta Brożek, Adam Stroiński, Anna Romaniak, Thierry Bourgoin

**Affiliations:** 1 Faculty of Natural Sciences, Institute of Biology, Biotechnology and Environmental Protection, University of Silesia in Katowice, Katowice, Poland; 2 Polish Academy of Sciences, Museum and Institute of Zoology of the Polish Academy of Sciences, Warsaw, Poland; 3 Institut de Systématique, Evolution, Biodiversité, ISYEB-UMR 7205 MNHN-CNRS-Sorbonne Université-EPHE-Univ. Antilles, Muséum National d’Histoire Naturelle, CP 50, 57 rue Cuvier, Paris, 75005 France

**Keywords:** Morphology, Metathoracic leg, Spiniform sensillum, Metatibiotarsal teeth

## Abstract

A review of the cuticular and sensory metatibial and metatarsal structures in cixiid planthoppers (Hemiptera: Fulgoromorpha) is proposed, depicting both their strong disparity and the great diversity of the patterns observed. Terminology and definitions for these structures are provided. The so-called lateral teeth of the metatibia in fact are particular styloconic sensory structures, called spiniform sensilla in Cixiidae. The apical metatibiotarsal teeth are non-sensory cuticular expansions, often bearing one or several chaetic sensilla ventrally, generally distributed in an internal and an external group of three teeth each, in some instances separated by a diastema; innermost and outermost teeth are generally larger. On the first tarsomere seven to eight teeth generally occur in one row, although two rows are observed in Brixidiini. A strong diversity of conformations and patterns is observed in the second metatarsomere. A specific subdorsal sensillum, of platellar type, may be present in the first metatarsomere teeth for a few taxa. It is generally present in the second metatarsomere, either as a narrow-based acutellar sensillum or as a broad-based platellar sensillum according to the taxon. Scanning electron microscope (SEM) analyses of 54 species of cixiids from all described tribes of the family, supplemented by data from the literature, are used to provide a metatibiotarsal diagnosis for each of the tribes of Cixiidae. In the state of our knowledge of the sufficiently precise observations of metatibiotarsal structures in the Cixiidae which are available, and of the phylogeny of the group as a frame of reference for their interpretations, we note that the observed patterns are probably the result of multiple and independent convergences and evolutionary regressions. These occurred at all levels of cixiid classification. Although these patterns can be useful in the identification of taxa at a low taxonomic level, they would be less useful for phylogenetic approaches.

## Introduction

The metathoracic legs are of primary importance in the taxonomy of hemipteran insects, bearing one of the oldest major characters recognized to separate Fulgoromorpha from Cicadomorpha (i.e., immobile vs. mobile metacoxa [1]). Additionally, the conformation of the second metatarsomere and the arrangements of its apical teeth have proved highly valuable in recognizing major divisions within planthoppers [2]. Emeljanov [3] has further highlighted the distinctive metatrochanter-femoral joint in planthoppers, particularly in the Cixiidae Spinola, 1839, and the arrangement of lateral metatibial spines, utilized in the classification of this family. According to this author, the conformation of some of these spines represents an apomorphic stage in certain cixiid tribes [3–7].

The current classification of Cixiidae relies heavily on Emeljanov’s contributions, as summarized in his ‘contribution to classification and phylogeny’ of the family [6] and Holzinger et al. [8]. However, even with obvious sampling biases, both morphology and molecular phylogeny tests by Ceotto and Bourgoin [9] and Ceotto et al. [10] failed to verify Emeljanov’s hypothesis. More recently and while awaiting new phylogenetic analyses, Luo et al. [11] proposed to simplify the approach to the problem by focusing first on the main lineages without necessarily applying formal taxonomy recognition, until a more complete and stable new system of classification closer to the phylogenetic results could be proposed. They thus identified three main cixiidian lineages, roughly covering the groups of tribes proposed by Emeljanov, namely the oecleinian, pentastirinian, and cixiinian lineages. In the recently published phylogenetic analysis [12] addressing the planthopper phylogeny and including 126 terminal taxa in the Cixiidae, these three lineages were well recovered and supported with the following groupings:

1. The oecleinian lineage, positioning as sister to the (pentastirinian + cixiinian) lineages, consists of all genera in Oecleini Muir, 1922, also including those of Bothriocerini Muir, 1923, poistionned as sister to the oecleini genus *Colvanalia* Muir, 1925, in contrast to a sister relationship between the two tribes as proposed by Emeljanov [6]; paraphyly of Oecelini was also previously confirmed by Le Cesne et al. [13];
2. The pentastirinian lineage including Borysthenini Emeljanov, 1989, sister to Pentastirini Emeljanov, 1971; and.
3. The cixiinian lineage consisting of all the other tribes, revealing the Cixiini Spinola, 1839 as a polyphyletic unit. Gelastocephalini Emeljanov, 2000 appeared sister to two main groups, the (Pintaliini Metcalf, 1938 + Chidaea^+^ clade (= Australian Cixiini genera)) group and the Andini^+^ group with the remaining cixiid tribes according to the following schema: (Andini Emeljanov, 2002 + (Eucarpiini Emeljanov, 2002 + (Bennini Metcalf, 1938 + (other Cixiini genera including Semonini Emeljanov, 2002)))). In Bucher et al.’s [12] analysis, the genus *Achaemenes* Stål, 1866 was placed in the cixiinian lineage sister to the Andini^+^ group. Additional reported results [14] placed Brixini Emeljanov, 2002 as sister to Andini, moved Gelastocephalini sister to Eucarpiini and *Achaemenes* as sister to all the cixiinian taxa. Two tribes remain unplaced: Bennarellini Emeljanov, 1989 and Brixidiini Emeljanov, 2002. The Mnemosynini Emeljanov, 1992, unfortunately sampled with only one species in Bucher et al. [12], were placed sister to the Oecleini in the oecleinian lineage.

**Fig. 1:**
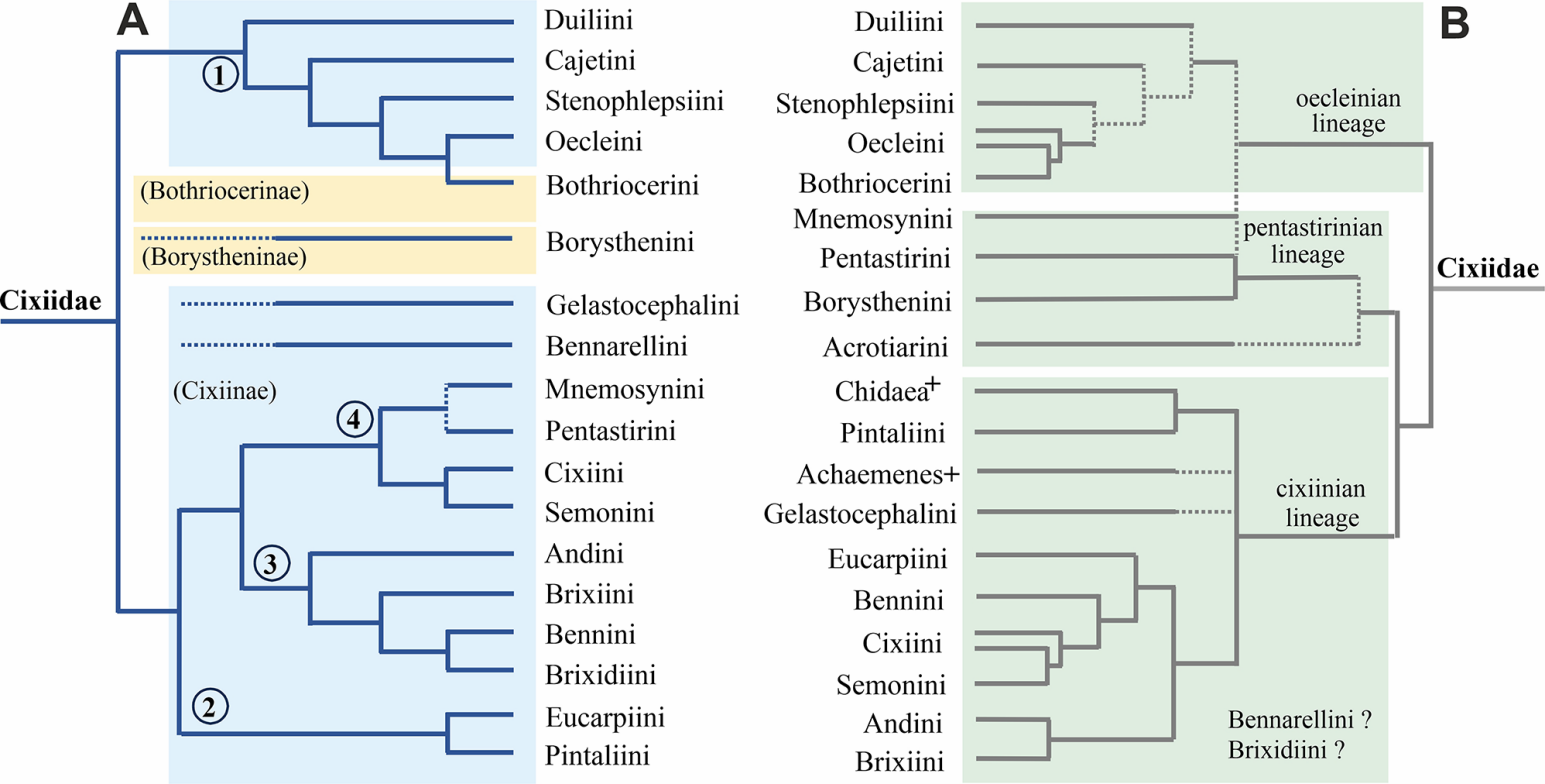
The presented hypothesis of relationships in the family Cixiidae is based on current and published phylogenetic data, which individually do not cover a complete set of tribes. **A** Redrawn and adapted from Emeljanov [6]. **B** Modified from the planthopper phylogeny of Bucher et al. [12], slightly modified according to Emeljanov [6], Luo et al. [11], and Bourgoin et al. [14]. Numbers in the circle refer to the main groups proposed by Emeljanov [6]. Dotted lines refer to uncertain potential relationships

Figure 1 shows both Emeljanov’s [6] proposed topology and the phylogeny of Bucher et al. [12], slightly modified with additional unpublished results of Bourgoin et al. [14], and following Emeljanov [6] for the tribes Duiliini Emeljanov, 2002, Cajetini Emeljanov, 2002, and Stenophlepsiini Metcalf, 1938, tentatively positioned in the oecleinian lineage.

Addressing the molecular phylogeny of Cixiidae is not inherently challenging; however, correlating the recovered clades with their own morphology poses a much more complex problem, in particular for providing identification keys and reliable classification based on morphology. Indeed, the morphological characters accessible to comparative analysis in Cixiidae are subject “to too many intermediate stages” [15] and “to a great homologous variability and reversals” [6], that are expressed within the 253 genera and 2625 species currently accounted in the family [16]. It results in a real “difficulty in producing a classification of the family” [2] taking into account morphologically well-recognizable natural groups as those newly disclosed by the molecular approaches. To try to overcome this problem, new character sets must be found, such as those previously tested with mouthpart structures [17]. With this objective, we aimed to investigate a new set of morphological data. Firstly, by examining the disparity of cuticular and sensory structures on the metathoracic legs of Cixiidae, including a morphological study of these structures, and secondly, by analyzing whether the distribution of these character states they disclose aligns with specific or shared patterns (potential morphological synapomorphies) among taxa. With this approach, we aimed to enhance the identification and diagnosis of the cixiid tribes and the newly identified lineages through molecular methods.

## Materials and methods

### Sampling

The analysis was approached at the tribal level by selecting species considered representative of their tribe or taxonomic units of equivalent rank, as they appear in the current classification of the family (Fig. 1). Well aware, however, of the limits of our approach, which cannot account for the internal homoplasy within the selected groups, we supplemented our data with those from the published literature when possible. Table 1 lists the sampling of the species used for the scanning electron microscope (SEM) observations, represented by 97 individuals from 54 species in 45 genera. For each taxon, its author and date of publication are provided the first time they are cited in the text. However, to avoid an excessively long list of references, we refer readers to the original description references found in the Fulgoromorpha Lists on the Web database [16].

**Table 1:** List of species observed with SEM

Tribe	Genus	Species
Duiliini Emeljanov, 2002	*Duilius* Stål, 1858	*D.* (*Duilius*) *tatianae* Emeljanov, 1964
	*Duilius* subgenus *Bitropis* Dlabola, 1985	*D.* (*B.*) *fasciatus* (Horváth, 1894)
Stenophlepsiini Metcalf, 1938	*Euryphlepsia* Muir, 1922	*E. vangoethemi* Van Stalle, 1985
Oecleini Muir, 1922	*Haplaxius* Fowler, 1904	*H. pictifrons* (Stål, 1862)
	*Myndus* Stål, 1862	*M. musivus* (Germar, 1825)
		*M. taffini* Bonfils 1983
	*Nymphocixia* Van Duzee, 1923	*N. unipunctata* Van Duzee, 1923
	*Nymphomyndus* Emeljanov, 2007	*N. caribbea* (Fennah, 1971)
	*Pinacites* Emeljanov, 1972	*P. calvipennis* (Emeljanov, 1972)
	*Trigonocranaus* Fieber, 1875	*T. emmeae* Fieber, 1876
	*Coframalaxius* Bourgoin & Le Cesne, 2022	*C. bletteryi* Le Cesne & Bourgoin, 2022
	*Meenocixius* Attié, Bourgoin & Bonfils, 2002	*M. virescens* Attié, Bourgoin &Bonfils, 2002
	*Mundopa* Distant 1906	*M. kotoshonis* Matsumura, 1914
	*Oecleus* Stål, 1862	*O. borealis* Van Duzee, 1912
Bothriocerini Muir, 1923	*Bothriocera* Burmeister, 1835	*Bothriocera* sp.
Mnemosynini Emeljanov, 1992	*Mnemosyne* Stål, 1866	*M. arenae* Fennah, 1945
Borysthenini Emeljanov, 1989	*Borysthenes* Stål, 1866	*B. maculatus* (Matsumura, 1914)
		*B. lacteus* Tsaur & Lee, 1987
Pentastirini Emeljanov, 1971	*Pentastira* Kirschbaum, 1868	*P. rorida* (Fieber, 1876)
	*Oecleopsis* Emeljanov, 1971	*O. artemisiae* (Matsumura, 1914)
	*Hyalesthes* Signoret, 1865	*H. luteipes* Fieber, 1876
	*Setapius* Dlabola, 1988	*Setapius* sp.
	*Reptalus* Emeljanov, 1971	*R. panzeri* (Löw, 1883)
		*R. quadricinctus* (Matsumura, 1914)
	*Oliarus* Stål, 1962	*O. annandalei* Distant, 1911
	*Pentastiridius* Kirschbaum, 1868	*P. beieri* (Wagner, 1970)
		*P. leporinus* (Linnaeus, 1761
	*Melanoliarus* Fennah, 1945	*M. complexus* (Ball, 1902)
		*M. kindli* Bourgoin, Wilson & Couturier, 1998
Cixiini (Achaemenes + clade)	*Achaemenes* Stål, 1866	*A. kalongensis* Synave, 1963
		*A. quinquespinosus* Synave, 1960
Cixiini (Chidaea^+^clade)	*Chidaea* Emeljanov, 2000	*Chidaea* sp.
Pintaliini Metcalf, 1938	*Pintalia* Stål, 1862	*P. vibex* Kramer, 1983
	*Muirolonia* Metcalf, 1936	*M. metallica* (Fowler, 1904)
	*Notocixius* Fennah, 1965	*N. helvolus* (Blanchard, 1852).
	*Cubana* Uhler, 1895	*Cubana* sp.
	*Monorachis* Uhler, 1901	*M. sordulentus* Uhler, 1901
Andini Emeljanov, 2002	*Andes* Stål, 1866	*A. marmoratus* (Uhler, 1896)
Brixiini Emeljanov, 2002	*Brixia* Stål, 1856	*Brixia* sp.
Gelastocephalini Emeljanov, 2000	*Gelastocaledonia* Löcker & Larivière, 2006	*G. monteithi* Löcker & Larivière, 2006
	*Wernindia* Löcker & Fletcher, 2006	*W. lorda* Löcker & Fletcher, 2006
Eucarpiini Emeljanov, 2002	*Eucarpia* Walker, 1857	*E. elisabethana* (Synave, 1962)
Bennini Metcalf, 1938	*Benna* Walker, 1857	*Benna* sp.
Cixiini Spinola, 1839	*Cixius* Latreille, 1804	*C. pini* Fitch, 1851
		*C. nervosus* (Linné, 1758)
	*Tachycixius* Wagner, 1939	*T. pilosus* (Olivier, 1791)
	*Macrocixius* Matsumura, 1914	*M. giganteus* Matsumura, 1914
	*Leptolamia* Metcalf, 1936	*L. radicula* Löcker, 2014
	*Cixiosoma* Berg, 1879	*C. bonaerense* Berg, 1883
Bennarellini Emeljanov, 1989	*Noabennarella* Holzinger & Kunz, 2006	*Noabenarella* sp.
Brixidiini Emeljanov, 2002	*Brixidia* Haglund, 1899	*B. boukokoensis* Synave 1980
		*B. variabilis* Van Stalle & Synave, 1984
Semonini Emeljanov, 2002	*Betacixius* Matsumura, 1914	*B. ocellatus* Matsumura, 1914
	*Kuvera* Distant, 1906	*K. tappanella* Matsumura, 1914

### Scanning electron microscope (SEM)

For the SEM studies, the third pair of legs were cut from dry specimens, manually cleared with a brush, and positioned on the handle with carbon tape. For observations of leg structures, a Phenom XL SEM with a backscatter electron detector (BSD) was used to collect signals from different interactions at the sample surface in a low vacuum chamber and kilovoltage (15 kV) without sample metallization. Images were captured with a microscope Phenom XL (Phenom-World, Eindhoven, The Netherlands) at the scanning microscopy laboratory, Faculty of Natural Sciences, University of Silesia, in Katowice, Poland. The magnification of the structures is represented in photos in the scale bar (µm).

### Terminology

A lexicon of all morphological terms with their description is provided in the first part of the results. When necessary for the pattern descriptions, sensilla and teeth were respectively numbered from proximal to distal extremity and from most external to most internal side. Many publications use the spelling and the adjectival form sensilla in mixed form, both Latin and English. In the present article, English descriptors of the sensillum types are used. These are acutellar, chaetic, platellar, spiniform, and styloconic sensillum/sensilla, respectively being *sensillum acutellaris*, *chaeticum*, *platellae*, *spiniformis*, and *styloconicum* in Latin singular; and *sensilla acutellarum*, *chaetica*, *platellarum*, *spiniformia*, and *styloconica* in Latin plural.

De Souza Amorim ‘s 1982 conventional notation [18] was used when necessary in naming some clades, with the support name of the clade being taken to be the basalmost taxon in the clade topology, i.e. clade A^+^ meaning clade A+(B+(C + D)).

## Results

### Diversity of morphological structures: character definitions

Cixiids exhibit three main types of metatibial cuticular structures: non-sensory cuticular spines, sensory setae or chaetic sensilla, and a new type of sensory structure called the spiniform sensillum, referring to their previous misidentification with spines [5]. For each of these structures, the following terminologies and definitions are applied.

**Fig. 2:**
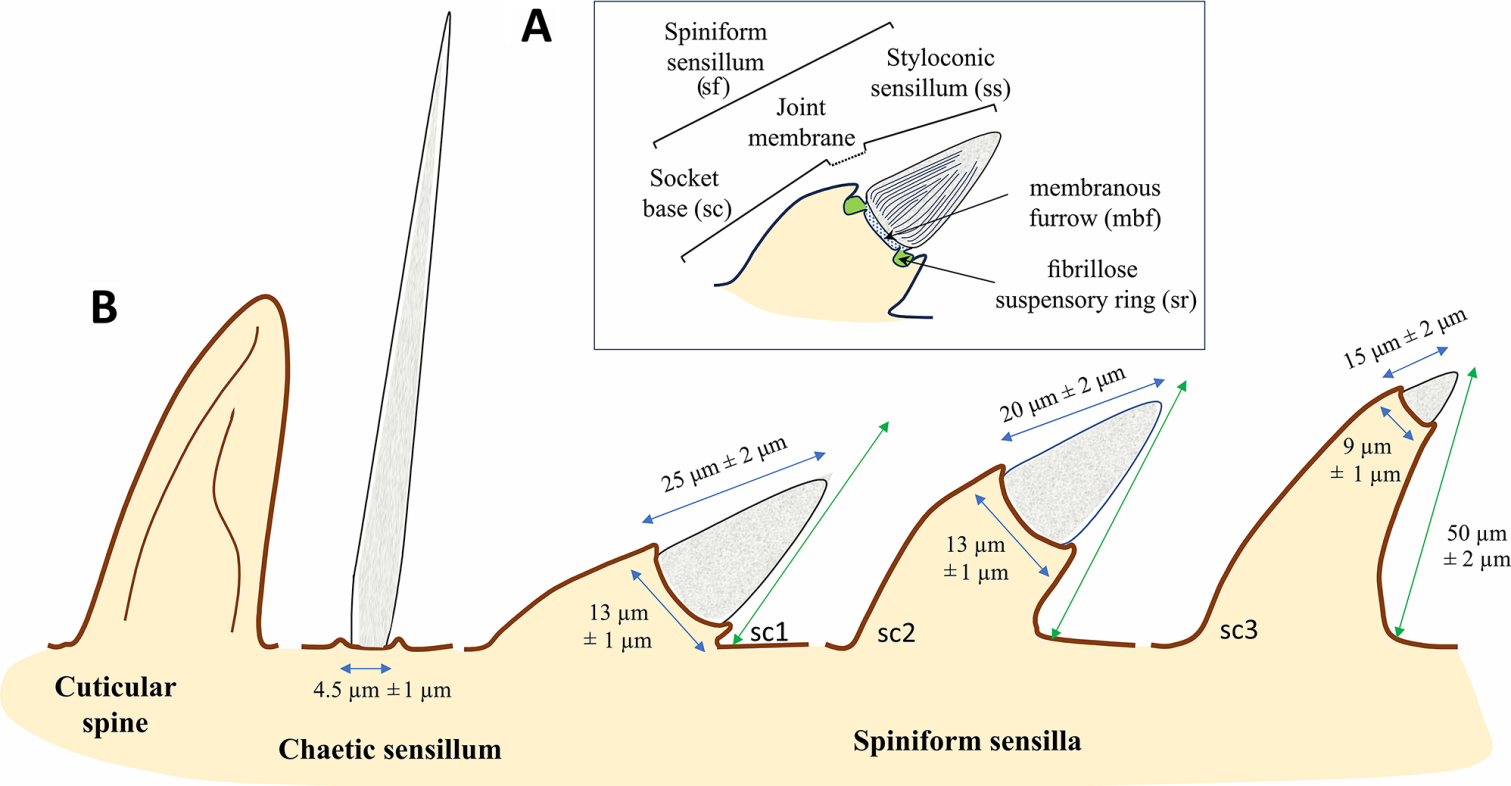
Morphological terminology of spiniform sensilla. **A** Lateral metatibial microstructures (sockets (sc) with the membranous furrow and sensillum). **B** Cuticular spine and socket types in Cixiidae. The length of the spiniform sensilla is marked with green arrows. Abbreviations: sc1, short base; sc2, medium cylindrical base; sc3, large base

**Fig. 3:**
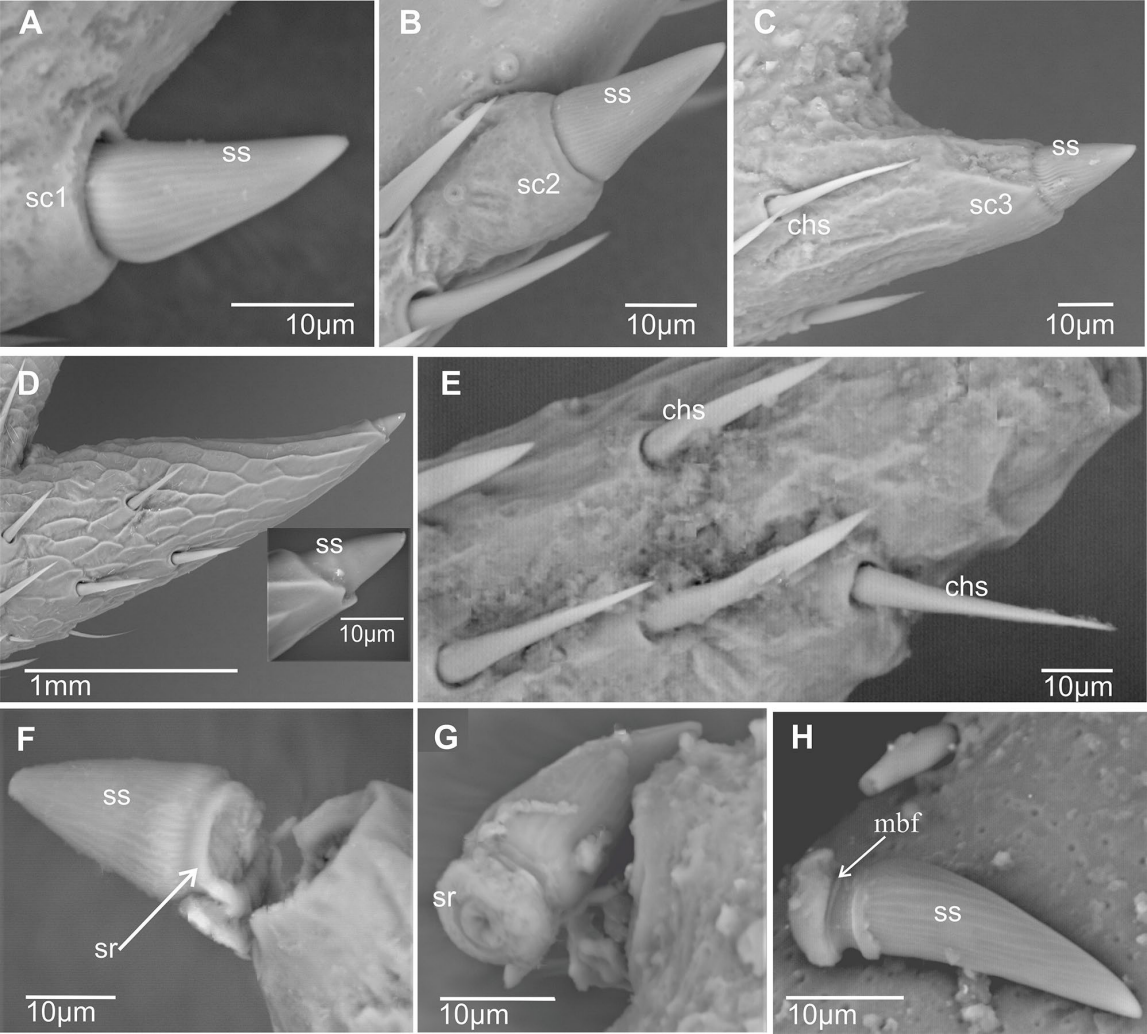
Spiniform and chaetic sensilla in some cixiid taxa. **A*** Bothriocera* sp. **B*** Pintalia vibex.***C*** Reptalus panzer*. **D*** Mnemosyne arenae.***E**,** F*** Pentastira rorida*. **G**,** H*** Borysthenes maculatus.* Abbreviations: chs, chaetic sensillum; mbf, membranous furrow; sc1, sc2, sc3, spiniform sensilla socket types; sr, suspensory ring; ss, styloconic sensillum sensitive part of the spiniform sensillum

**Metatibial spiniform sensillum (sf)** (Figs. 2 and 3). A peculiar type of styloconic sensillum (ss) carried by a distinctive cylindrical basal cuticular socket (sc) of an insensitive cuticle, more or less projected over the surface of the metatibia, probably of mechanosensory function (definition adapted from Shields [19]). Spiniform sensilla are specialized structures, developed in the outer lateral side of the metatibia. So far, such sensilla are only known in Cixiidae, although they are secondarily absent in a few taxa in the family. Three main types of spiniform sensilla are observed according to the size of the basal socket: (1) short base (sc1), elongated on the metatibial surface, no longer than the sensillum (Figs. 2 and 3A); (2) medium cylindrical base (sc2), no longer than the sensillum, distinctly erected above the metatibial surface (Figs. 2 and 3B); and (3) large cylindrical base (sc3), much longer than the sensillum, erected and strongly developed over the metatibia surface (Figs. 2 and 3C and D).

Whatever the type, the sensory part (= styloconic sensillum) exhibits an elongated conical shape, more or less twice longer than wide, with a finely longitudinally wrinkled shaft (Figs. 2A and 3A − C). The shorter the socket base, the longer the styloconic sensillum, the latter with a mean of 25 μm (long), 20 μm (medium), and 15 μm (short), respectively, for each type of sensillum (Fig. 2). Fine irregular wrinkles adorn large basal sockets (Figs. 2 and 3C and D). Short and medium-sized basal socket bases support wider styloconic sensilla of 13 μm ± 0.1 μm diameter, while they appear distinctly smaller (9 μm ± 0.1 μm) when supported by large-sized basal sockets. Often, the size of the basal socket increases from the proximal to the distal parts of the metatibia. The total length of the spiniform sensilla is marked with green arrows in Fig. 2B. At its proximal end, the styloconic sensillum shaft (ss) connects to its base by a joint membrane (*sensu* Keil & Steinbrecht [20]) materialized by a narrower membranous furrow (mbf) followed by a roughly fibrillose ring called the suspensory ring (sr) (Figs. 2 and 3F − H). The suspensory ring connects the shaft’s base with the surrounding cuticle of the metaleg allowing some flexibility of sensillum.

**Chaetic sensilla** These are conventional mechanosensory sensilla characterized by their sharp-tipped structure, which is inserted into a distinctive basal flexible ringed socket. They exhibit variation in length and often display cuticular sculpturing [21]. Irregularly distributed across the surface of the metaleg, these sensilla are frequently aligned proximo-distally. In some instances, they are found at the base of the large cylindrical sockets of the spiniform sensilla (Fig. 3D, E).

**Fig. 4:**
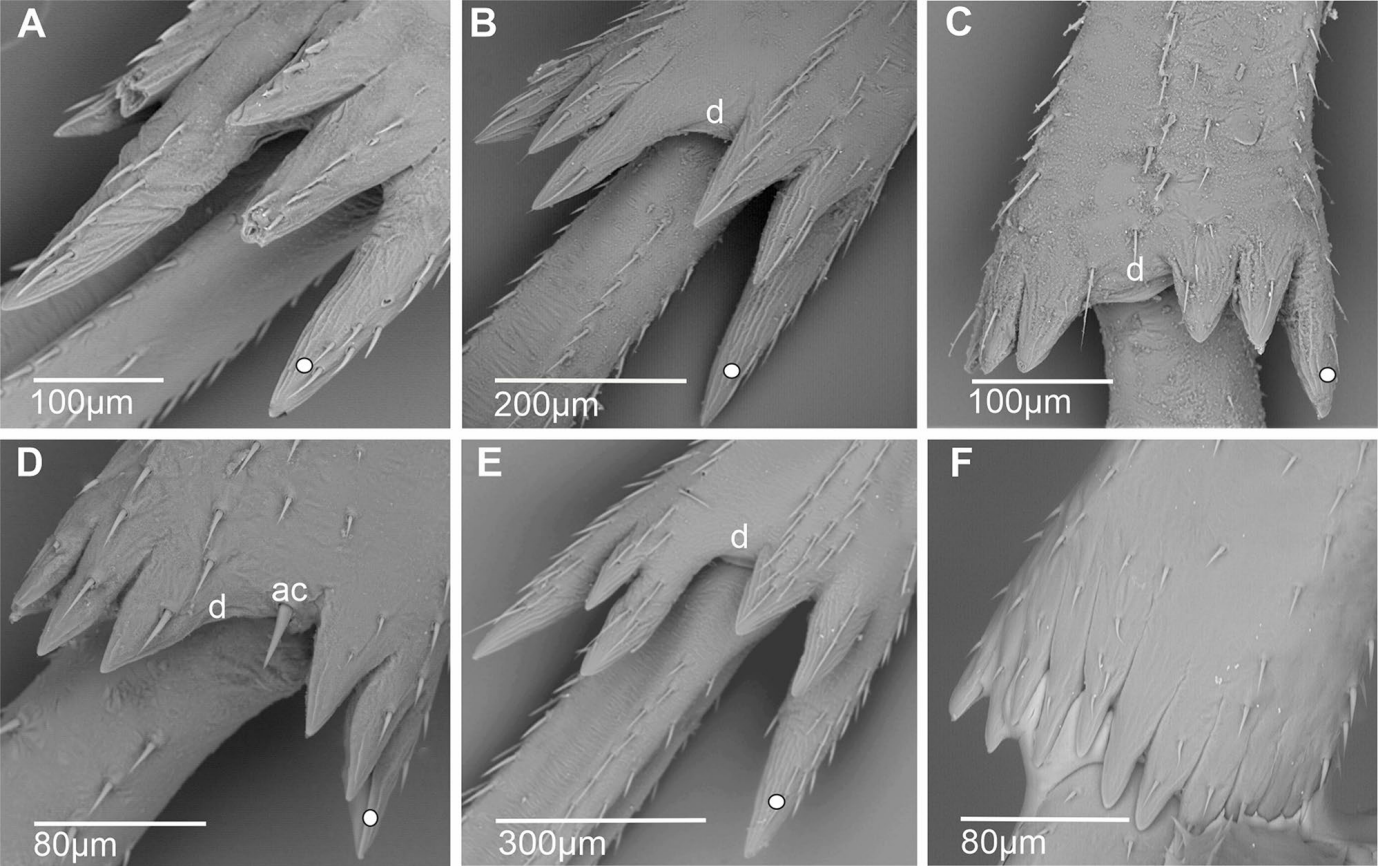
Conformation of metatibial apical teeth in some Cixiidae (ventral view). **A*** Benna* sp. **B*** Cixius nervosus*. **C*** Chidaea* sp. **D*** Bothriocera* sp. **E*** Mnemosyne arenae*. **F*** Euryphlepsia vangoethemi*. White dot indicates outermost external tooth. Abbreviations: ac, acutellar sensillum; d, metatibial diastema

**Apical metatibial teeth** (Fig. 4). This refers to a set of flattened, tooth-like cuticular projections with non-sensory functions located at the apex of the metatibia. The apical teeth are distributed into two groups, internal and external, each consisting of three teeth, representing the presumed plesiomorphic state. A diastema (d), more or less extended, may separate these two groups. The external tooth in each group is often equal to or more developed than the others, with the outermost tooth of the external group being consistently larger, except in Bennini, where it is the innermost tooth of the internal group (Fig. 4A); a derived conformation is observed in Stenophlepsiini (Fig. 4F). Some taxa show variations such as the reduction or duplication of the number of teeth, ranging from five to seven teeth.

Notably, in all existing planthoppers families chaetic sensilla and sarcosetae seem to be absent on their dorsal side. However, sarcosetae are present in a few other Cretaceous fossil families, i.e. Jubisentidae Zhang, Ren & Yao, 2019, Katlasidae, Luo, Jiang and Szwedo, 2020, Lalacidae, Hamilton, 1990’s tribes (Lalacini Hamilton, 1990, Protodelphacini Hamilton, 1990) and Perforissidae Shcherbakov, 2007 [22, 23].

**Metatibial diastema (d)** (Fig. 4). External and internal groups of apical metatibial teeth might be separated by more or less wide space, diastema (d). It might be present and wider than a tooth base width, distinctly shorter, or absent. In instances where the diastema is present, it is devoid of setae or may support an acutellar sensillum (ac), notably observed in Bothriocerini (Fig. 4D).

**Fig. 5:**
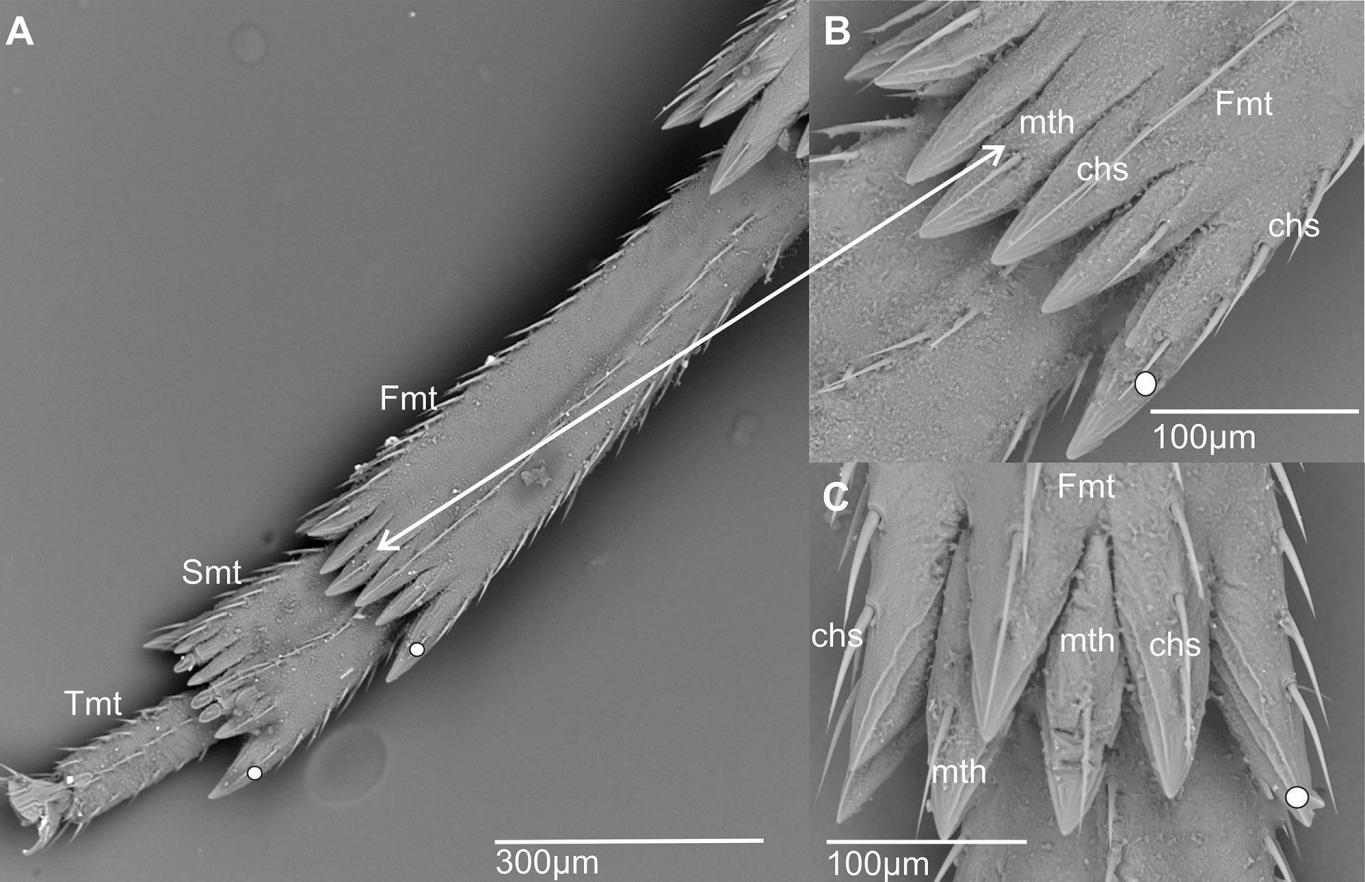
Shape of the metatarsus and arrangement of the metatarsal teeth (ventral view). **A**, ** B*** Pintalia vibex*. **C*** Brixidia boukokoensis*. White dot indicates outermost external tooth. Abbreviations: chs, chaetic sensillum; Fmt, first metatarsomere; mth, metatarsal tooth; Smt, second metatarsomere; Tmt, third metatarsomere

**Metatarsomere** (Fig. 5) Planthopper metatarsus is divided in three metatarsomeres followed apically by a pair of ungues and a median arolium [24–26]. In Cixiidae, the first metatarsomere (Fmt) or basitarsomere is, at least, twice longer than the second one (Smt) (Fig. 5A).

**Fig. 6:**
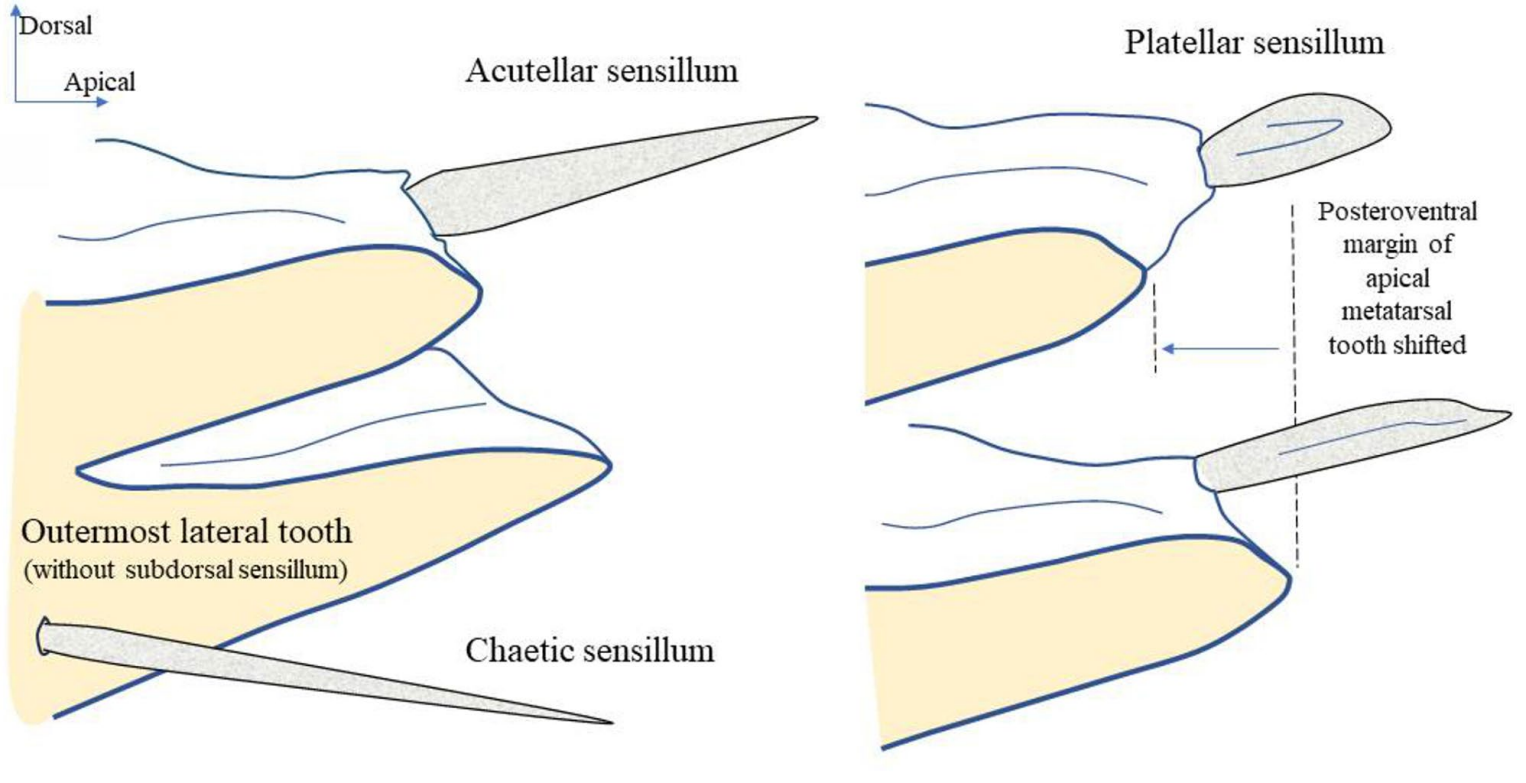
Schematic representation of apical metatarsal teeth types in Cixiidae, with subdorsal sensilla types acutellar and platellar. Chaetic sensilla occur only on the ventral side

**Metatarsal teeth (mth)** (Figs. 5 and 6). A series of flattened, tooth-like cuticular projections of non-sensory function at the apex of the first and second metatarsomere, sometimes erroneously called spines. They are arranged in one arched row such as in *Pintala vibex* (Fig. 5B) or exceptionally in two rows such as in *Brixidia* Haglund, 1899 (Fig. 5C). The number of these projections varies among taxa, and notably, they are absent on the third metatarsomere.

Metatarsal teeth look proportionally slightly longer than the apical metatibial teeth due to the deeper incisions between them. In all cixiid species, the ventral margin of the tooth is generally well developed, and each metatarsal tooth bears one or several ventral subapical chaetic sensilla (Fig. 5). As a cuticular projection of the metatarsomere, the ventral side of the tooth is always well sclerified, while the dorsal side appears more membranous, unexposed, and in some instances carries an additional subdorsal sensillum. In the second metatarsomere, the ventral side of the tooth might become reduced, often marked apically by a thin whitish band in SEM photos. This reduction fully exposes the subdorsal sensillum when present (Fig. 6). On each side, the innermost and outermost metatarsal teeth always lack a subdorsal sensillum.

**Subdorsal sensilla of metatarsal teeth.** They have been particularly studied by Emeljanov [25], who categorized them into two main types: typical chaetic sensilla (further divided into three subtypes of simple, shortened, and blunt setae) and sarcosetae divided into acutellar sensilla and platellar sensilla. Notably, these subdorsal chaetica sensilla appear to be absent in the second metatarsomere, on which only acutellae or platellae have been observed.

**Fig. 7:**
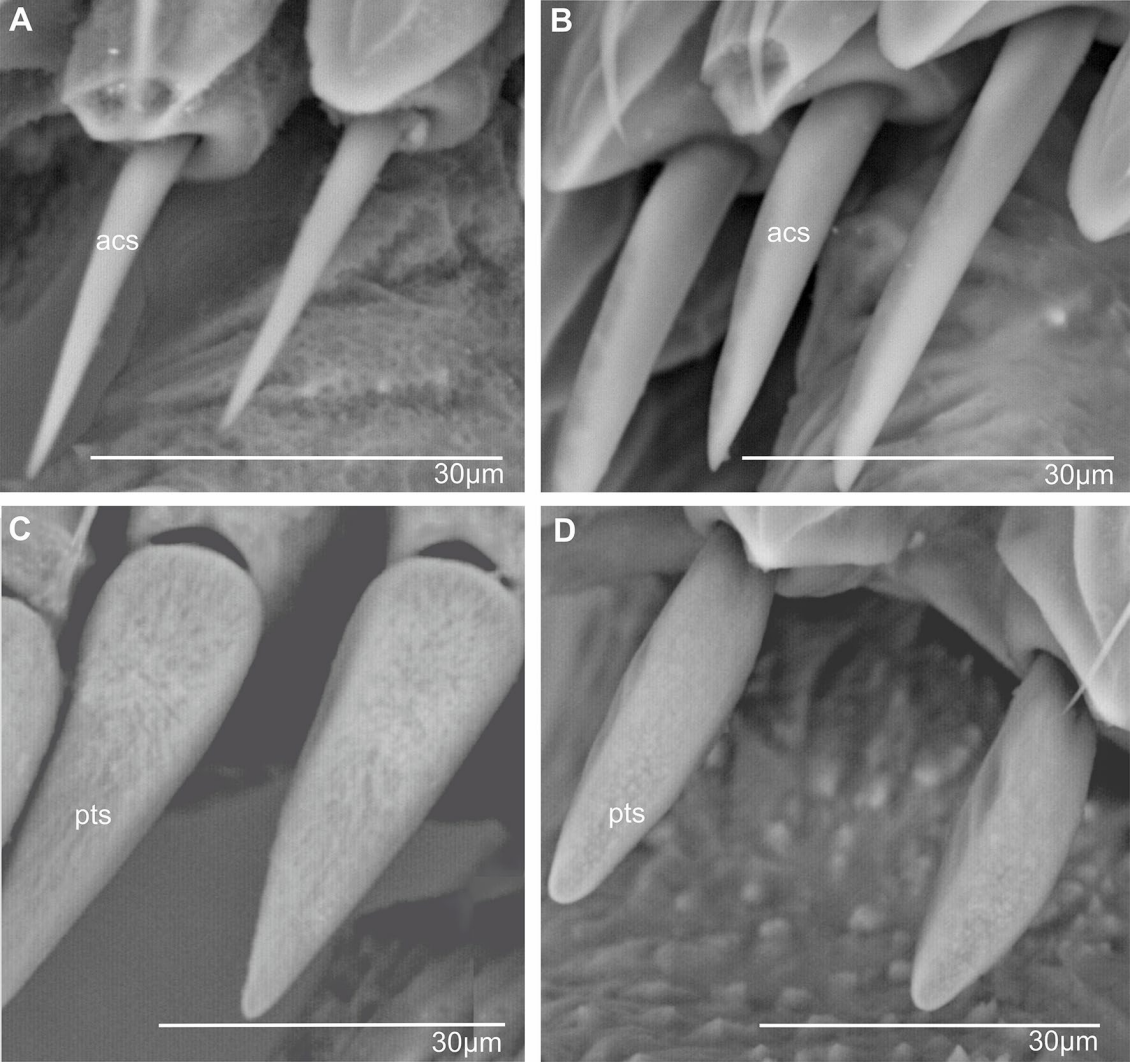
Shape of subdorsal acutellar and platellar sensilla in some Cixiidae. **A*** Bothriocera* sp. **B*** Nymphomyndus caribbea*. **C*** Setapius* sp. **D*** Cixius nervosus*. Abbreviations: Acs, acutellar sensillum; pts, platellar sensillum

**Acutellar sensilla** (Figs. 6 and 7). These sensilla are modified chaetic sensilla, similarly shaped. They are however more robust, 30–50 μm long, straight, wider proximally and apically tapered. Their cross-section is rounded to slightly triangular and compressed laterally (Fig. 7B); however, acutellar sensilla are distinctly narrower than their basal socket (Fig. 7A, B).

**Platellar sensilla** (Figs. 6 and 7). These modified acutellar sensilla, 30–40 μm in length, are characterized by their shorter, thicker, and more swollen structure compared to typical acutellar sensilla. Their section is approximately as wide as the supporting socket. They exhibit various forms ranging from an elongated, robust and swollen cone, wider proximally, with a more or less rounded section and lacking ridges (Fig. 7C), to a more dorso-ventrally compressed sensilla. This latter type is wider around mid-length with a roughly triangular section slightly ridged as observed in *C. nervosus* (Fig. 7D).

### Diversity of the morphological structures: distribution and patterns

**Fig. 8:**
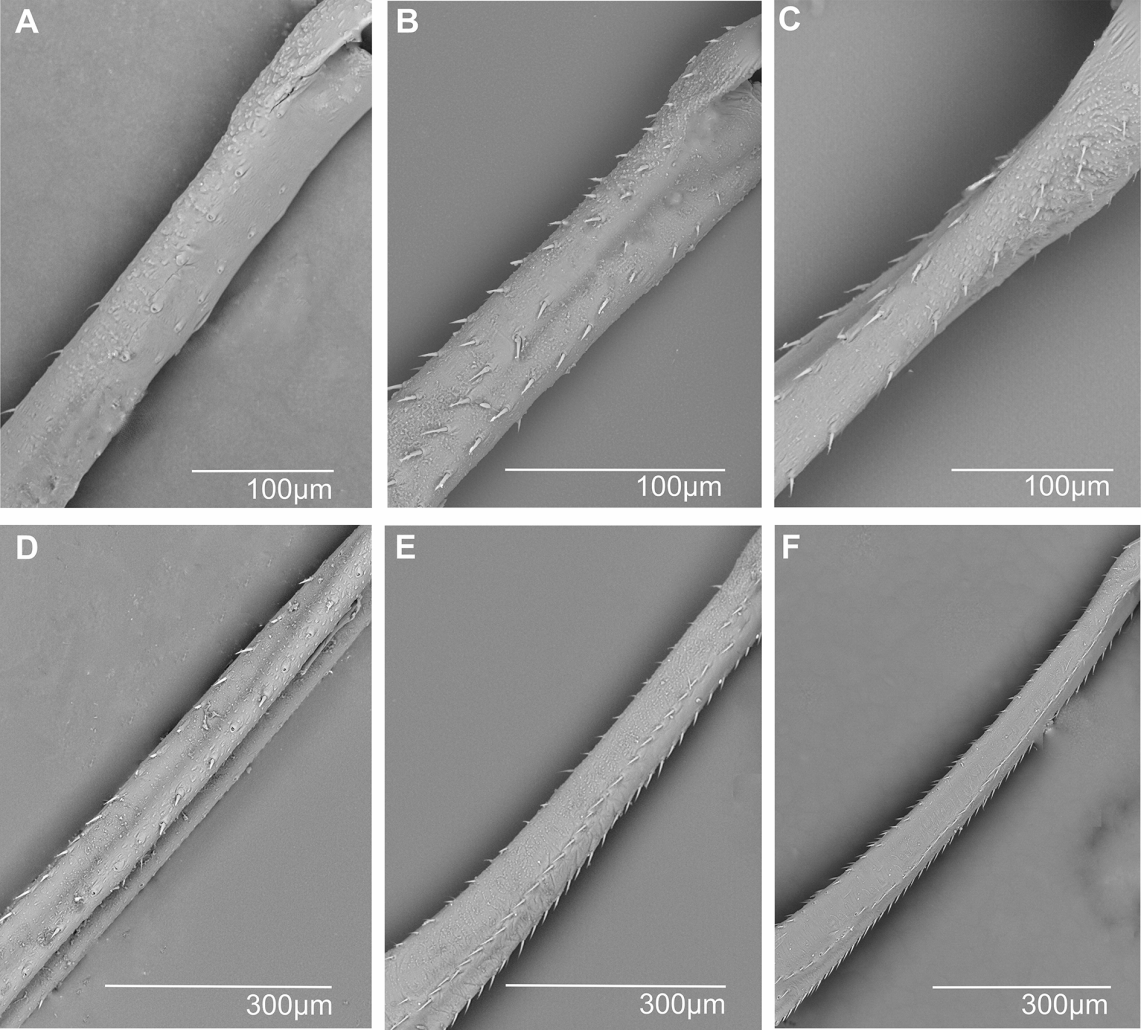
Distribution and shape of the lateral sensilla structures on the tibia in Oecleini. **A** *Duilius* (*Duilius*) *tatianae*. **B** *Haplaxius pictifrons*. **C** *Myndus musivus*. **D** *Muirolonia metallica*. **E** *Leptolamia radicula*. **F** *Achaemenes quinquespinosus*

**Metatibial spiniform sensilla** Most often referred to as lateral tibial spines, we report here a specialized type of styloconic sensilla carried by a distinctive basal cylindrical cuticular socket more or less projecting over the surface of the metatibia, that we propose to call spiniform sensilla. So far, they have been only observed in the Cixiidae, and they are notably absent in their sister family Delphacidae. However, they were not observed in all cixiid taxa and they were absent: (1) in most taxa of the oecleinian lineage as in the tribes Duiliini (such as *Duilius* (*Duilius*) *tatianae* (Fig. 8A), although present in the other Duiliini subgenus *Duilius* (*Bitropis*)), Stenophlepsiini (such as in *Euryphlepsia vangoethemi*), and in most Oecleini taxa: *Haplaxius pictifrons* (Fig. 8B), *Myndus musivus* (Fig. 8C), *Nymphocixia unipunctata*,* Nymphomyndus caribbea*,* Pinacites calvipennis*,* Trigonocarnaus emmeae*,* Coframalaxius bletteryi*,* Meenocixius virescens*; and (2) in the cixiinian lineage in the tribes Pintalini (*Muirolonia metallica* (Fig. 8D)), Eucarpiini (*Eucarpia elisabethana*), Cixiini (*Leptolamia radicula* (Fig. 8E)), and in the separated clade of the genus *Achaemenes* (*A. kalongensis* and *A. quinquespinosus* (Fig. 8F)).

**Fig. 9:**
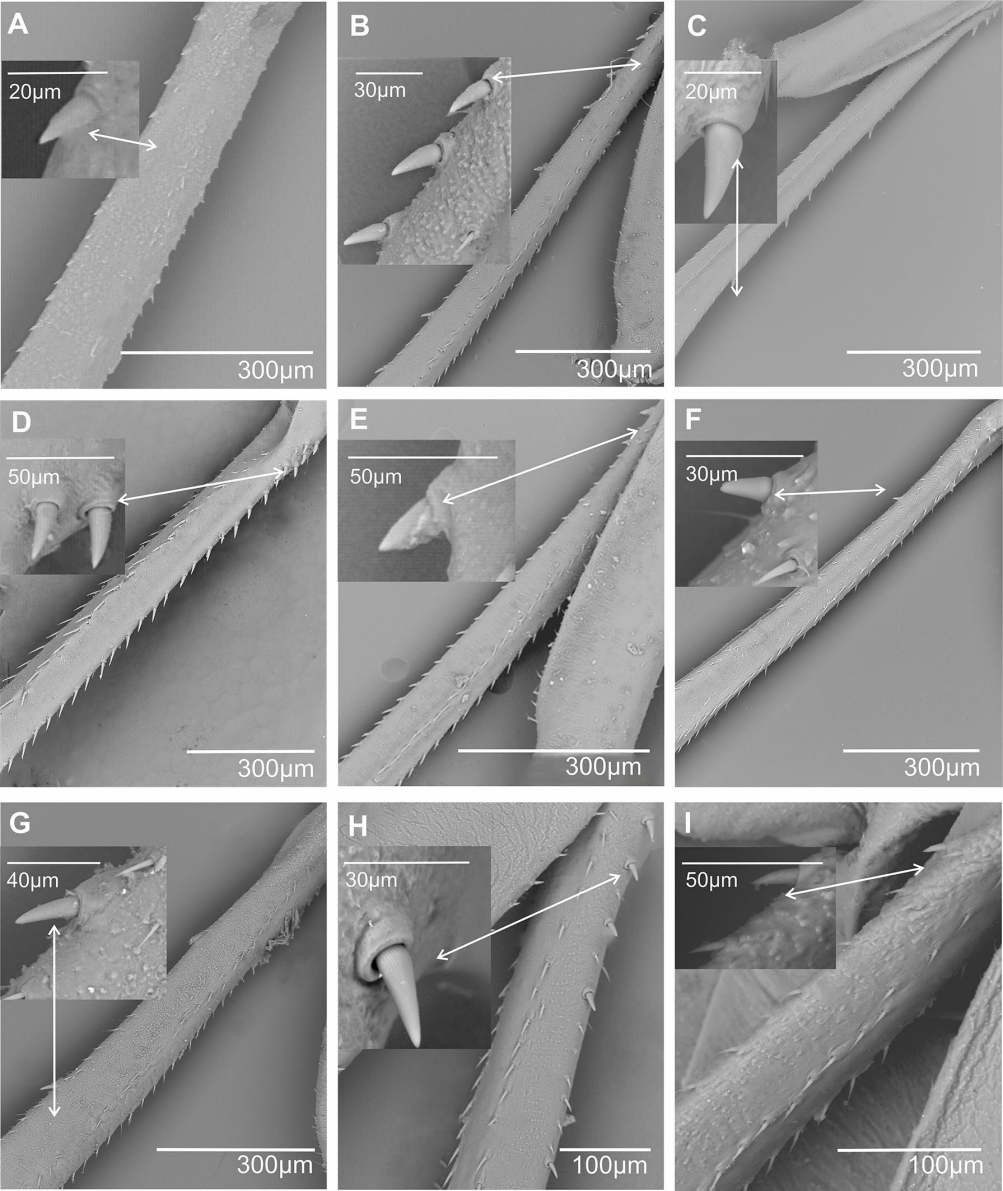
Distribution and shape of the lateral spiniform sensilla on the tibia. **A** *Benna* sp. **B** *Borysthenes lacteus*. **C** *Bothriocera* sp. **D** *Brixidia boukokoensis*. **E** *Brixia* sp. **F** *Notocixius helvolus*. **G** *Chidaea* sp. **H** *Noabennarella* sp. **I** *Gelastocaledonia monteithi*

**Fig. 10:**
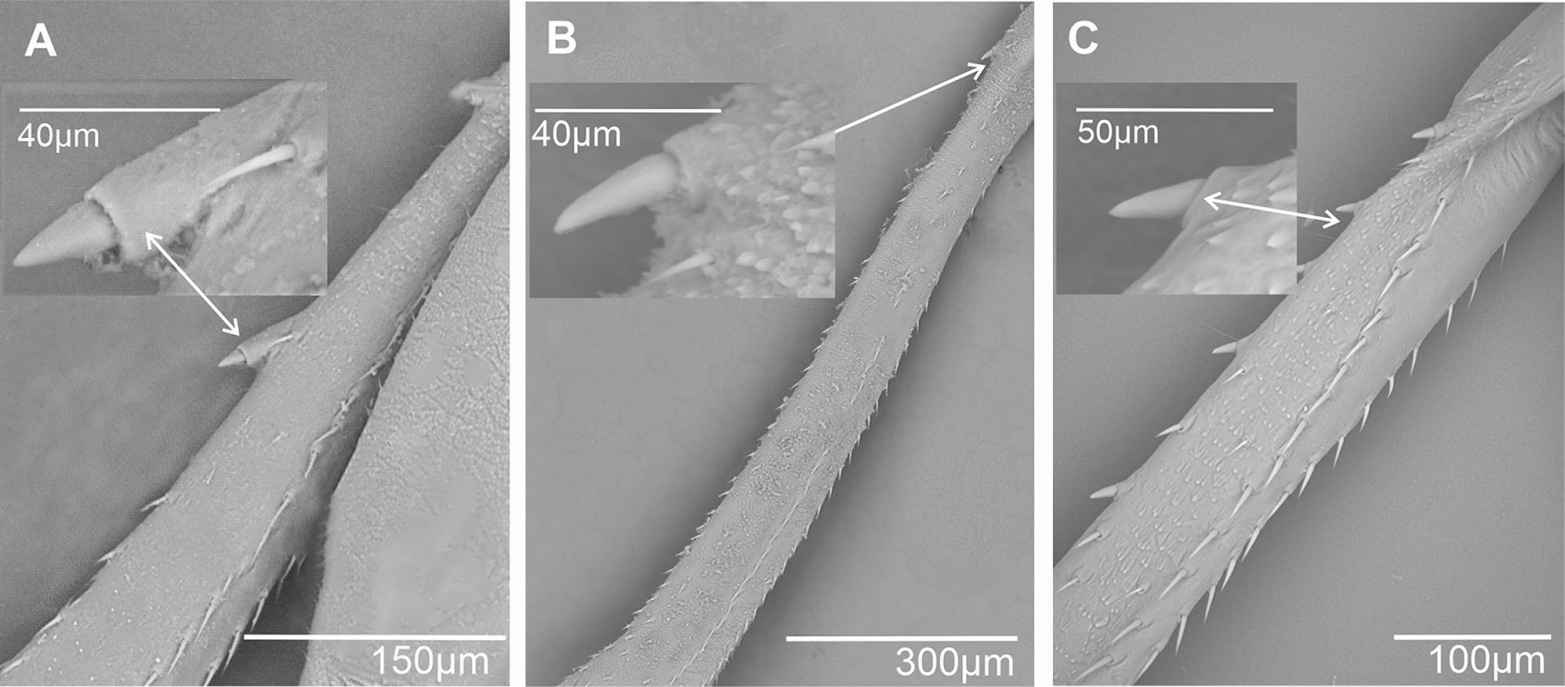
Distribution and shape of the lateral spiniform sensilla on the tibia. **A*** Duilius* (*Bitropis*) *fasciatus*. **B*** Mundopa kotoshonis*. **C*** Oecleus borealis*

**Fig. 11:**
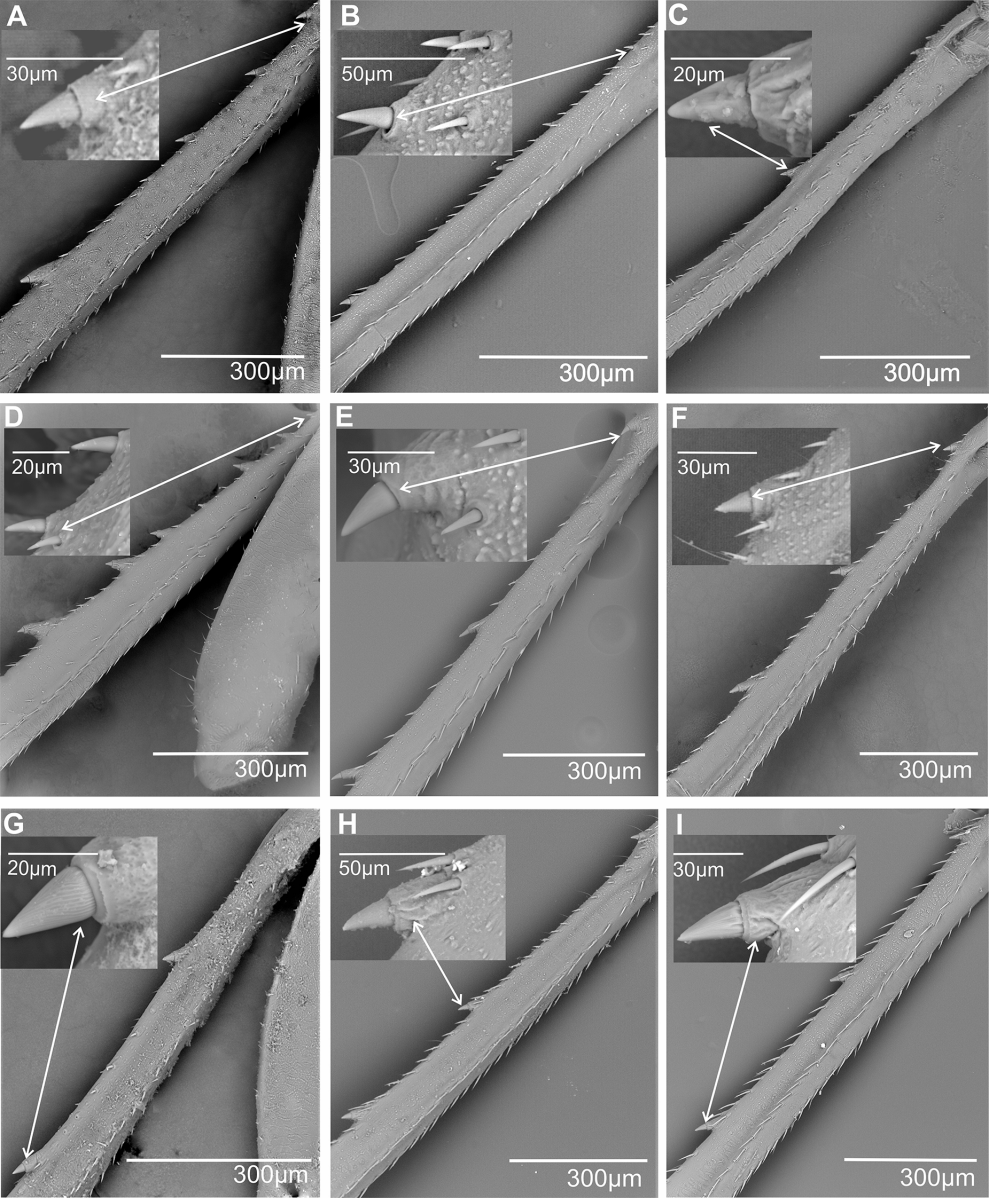
Distribution and shape of the lateral spiniform sensilla on the tibia. **A** *Macrocixius giganteus*. **B** *Andes marmoratus*. **C** *Hyalesthes luticeps*. **D** *Oecleopsis artemisiae*. **E** *Cixius pini*. **F** *Tachycixius pilosus*. **G** *Cubana* sp. **H** *Pintalia vibex*. **I** *Monorachis sordulentus*

**Fig. 12:**
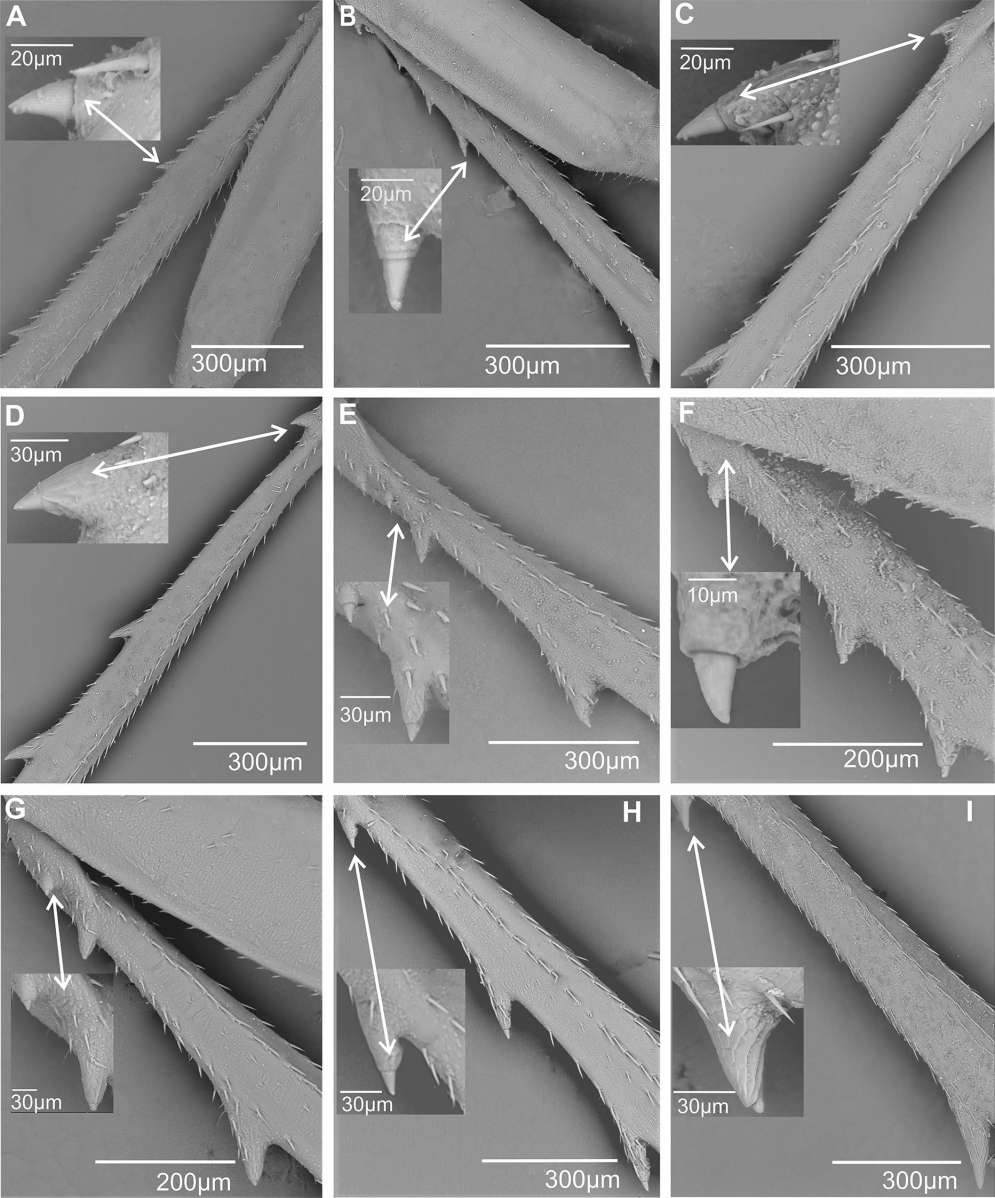
Distribution and shape of the lateral spiniform sensilla on the tibia. **A** *Betacixius ocellatus*. **B** *Kuvera tappanella*. **C** *Cixius nervosus*. **D** *Cixiosoma bonaerense*. **E** *Melanoliarus complexus*. **F** *Pentastiridius beieri*. **G** *Oliarus annandalei*. **H** *Pentastira rorida*. **I** *Mnemosyne arenae*

In all other cixiid taxa, spiniform sensilla were observed according to five main patterns:

1) Only small-sized spiniform sensilla with short base (sc1) are present in various numbers. Eleven of them are regularly distributed up to the 2/3 distal lateral side of the tibia (11sc1) in *Benna* sp. (Fig. 9A) or distributed in two groups: a basal proximal one and a more distal one, (3sc1 + 6sc1) in *Borysthenes lacteus* (Fig. 9B) and (2sc1 + 9sc1) in *Bothriocera* sp. (Fig. 9C). In *Brixidia boukokoensis* (Fig. 9D), three pairs of spiniform sensilla are observed proximally, followed by five singular ones extending in the first 1/3 of the metatibia (3 × 2sc1 + 5sc1). In most species, the number and distribution of these short-sized sensilla vary according to the genera in the tribes: (2sc1) in *Brixia* sp. (Fig. 9E), *Notocixius helvolus* (Fig. 9F), and *Chidaea* sp. (Fig. 9G); (5sc1) in proximal half part of the metatibia in *Noabennarella* sp. (Fig. 9H); and only one proximal (1sc1) in Gelastocephalini *Wernindia lorda* and *Gelastocaledonia monteithi* (Fig. 9I). In some Oecleini taxa, two small spiniform sensilla with a short base (sc1) are observed in *Duilius* (*Bitropis*) *fasciatus* (Fig. 10A), and *Mundopa kotoshonis* (Fig. 10B); *Oecleus borealis* has five such structures (Fig. 10C).

2) Spiniform sensilla with short (sc1) and medium-sized (sc2) sockets are distributed in various configurations: (3sc1 + 1sc2) before the middle of the metatibia in the Cixiini *Macrocixius giganteus* (Fig. 11A), (5sc1 + 1sc2) in the Andini *Andes marmoratus* (Fig. 11B), and in the Pentastirini *Hyalesthes luticeps* (Fig. 11C) extending on the 2/3 proximal part of the metatibia. Eight spiniform sensilla increasing in size are observed in the Pentastirini *Oecleopsis artemisiae* (Fig. 11D), up to the distal end of the metatibia.

3) Only medium-sized spiniform sensilla with base sockets (sc2) were observed: (3sc2) before mid-metatibia in Cixiini *Cixius pini* (Fig. 11E), but on the 3/4 on the metatibia in *Tachycixius pilosus* (Fig. 11F) and in all Pintalini species: *Cubana* sp. (Fig. 11G), *Pintalia vibex* (Fig. 11H), and *Monorachis sordulentus* (Fig. 11I). In Semonini, four spiniform sensilla (4sc2) were found in *Betacixius ocellatus* (Fig. 12A) and up to six (6sc2) in *Kuvera tappanella* (Fig. 12B).

4) In a few Cixiini and Pentastirini taxa a mix of medium- and large-sized (sc3) spiniform sensilla is observable, such as in *Cixius nervosus* (Fig. 12C) with the formula (1sc2 + 2sc3) not surpassing mid-metatibia, or up to its distal part in *Cixiosoma bonaerense* (Fig. 12D). The pattern (2sc2 + 2sc3) was observed in *Melanoliarus complexus* (Fig. 12E) and *Pentastiridius beieri* (Fig. 12F) reaching the middle length of the metatibia.

5) Taxa of the pentastirinian lineage including in the Mnemosynini exhibiting only the large type one. For instance, three large spiniform sensilla (3sc3) increasing in size toward the distal part of metatibia are present in *Oliarus annandalei* (Fig. 12G), *Pentastira rorida* (Fig. 12H), *Reptalus panzeri*, *Setapius* sp., and two largeones (one proximal and one in the 2/3 distal) in *Mnemosyne arenae* (Fig. 12I).

**Apical metatibial teeth**. Apical metatibial teeth are of wide occurrence in planthoppers. There are without sensory function, although they use to carry ventral chaetica sensilla in Cixiidae. The plesiomorphic condition [6] is probably a set of an internal and an external group of three teeth each separated or not by a deeper, dorsally rounded incision, looking as a narrow diastema, or by a distinctly dorsally transverse wide diastema (Table 2).

**Table 2:** Presence or absence of the diastema in Cixiidae tribes and selected taxa

Diastema	Taxa
Narrow	Cixiini (*Cixius*,* Tachycixius*) Pentastirini (*Oecleopsis*,* Pentastira*,* Pentastiridius*,* Reptalus*)
Wide	Bothriocerini (bearing one acutellar sensillum), Gelastocephalini (*Gelastocaledonia*), Pintalini (*Notocixius*), ‘Australian Cixiini clade’ (*Chidaea* sp.), Pentastirini (*Setapius*), Oecleini, Mnemosynini
Absent	Acrotiarini, Borysthenini, Andini, Bennarellini, Bennini, Brixidiini, Brixiini, Cixiini (*Leptolamia*,* Macrocixius*,* Cixiosoma*), *Achaemenes*, Gelastocephalini (*Wernindia)*, Duiliini, Eucarpinii, Pintalini (*Cubana*,* Pintalia*,* Monorachis*,* Muirolonia*), Semonini (*Betacixius*,* Kuvera*), Stenophlepsiini, Pentastirini (*Oliarus*,* Melanoliarus*,* Hyalesthes*)

**Fig. 13:**
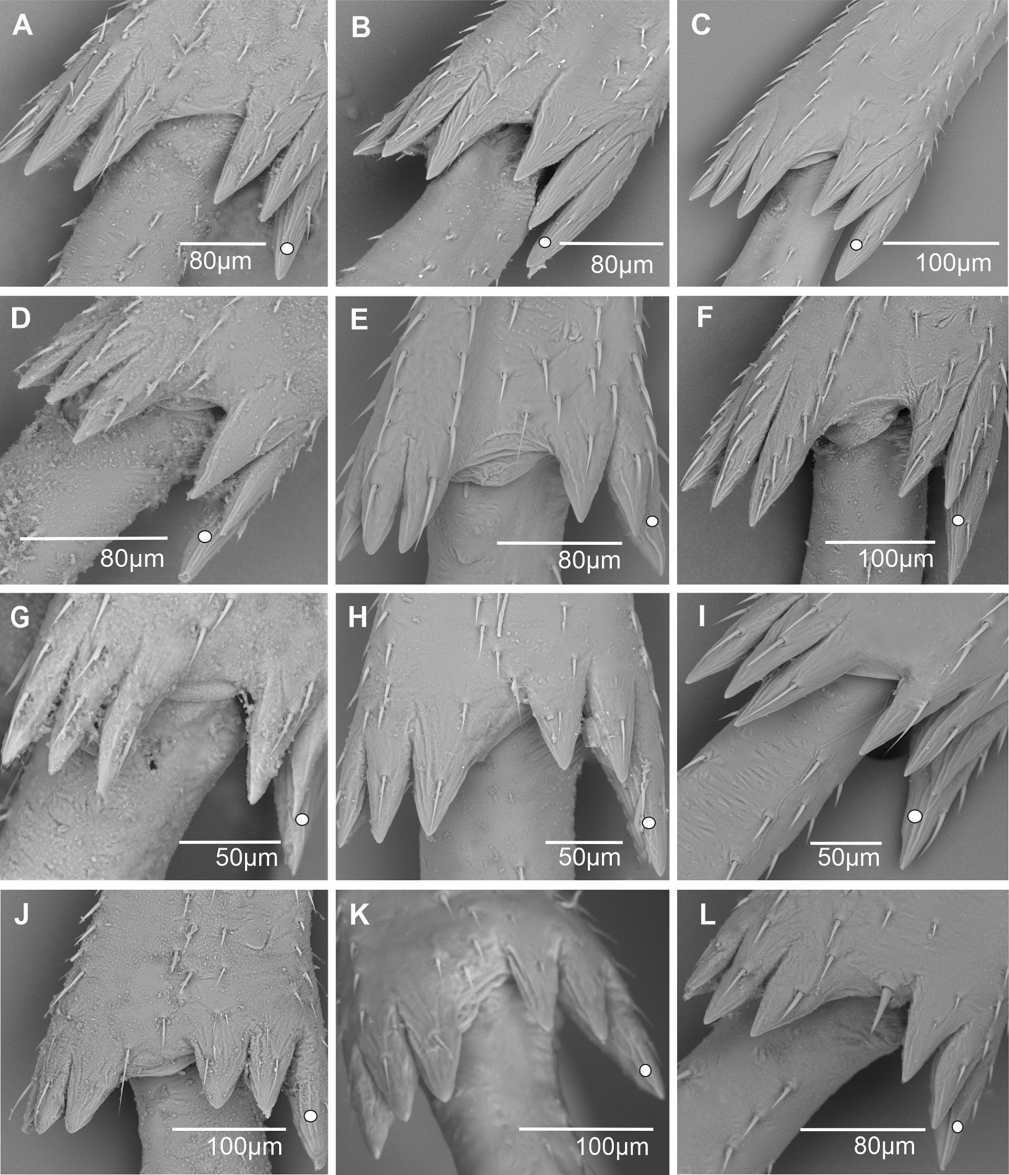
Distribution and types of the apical teeth on the tibia (ventral view). White dot indicates outermost external tooth. **A*** Haplaxius pictifrons*. **B*** Myndus taffini*. **C*** Nymphomyndus cribbea*. **D*** Mundopa kotoshonis*. **E*** Oecleus borealis*. **F*** Pinacites calvipennis*. **G*** Coframalaxius bletteryi*. **H*** Notocixius helvolus*. **I*** Setapius* sp. **J*** Chidaea* sp. **K*** Gelastocaledonia monteithi*. **L*** Bothriocera* sp

In most cixiid taxa of our study, the diastema was absent. It was however typically present as a wide diastema in all Oecleini—*Haplaxius pictifrons* (Fig. 13A), *Myndus taffini* (Fig. 13B), *M. musivus*,* Nymphomyndus cribbea* (Fig. 13C) *Nymphocixia unipunctata*, *Mundopa kotoshonis* (Fig. 13D), *Oecleus borealis* (Fig. 13E), *Trigonocranus emmeae*, *Pinacites clavipennis* (Fig. 13F), and *Coframalaxius bletteryi* (Fig. 13G)—and in Bothriocerini, Pintaliini *Notocixius helvolus* (Fig. 13H), Pentastirini *Setapius* sp. (Fig. 13I), Cixiini *Chidaea* (Fig. 13J), and Gelastocephalini *Gelastocaledonia monteithi* (Fig. 13K). In the oecleinian lineage the diastema is notably absent in Duiliini and Stenophlepsiini (Fig. 4F). In Bothriocerini the wide diastema also bears a single acutellar sensillum (Fig. 13L).

**Fig. 14:**
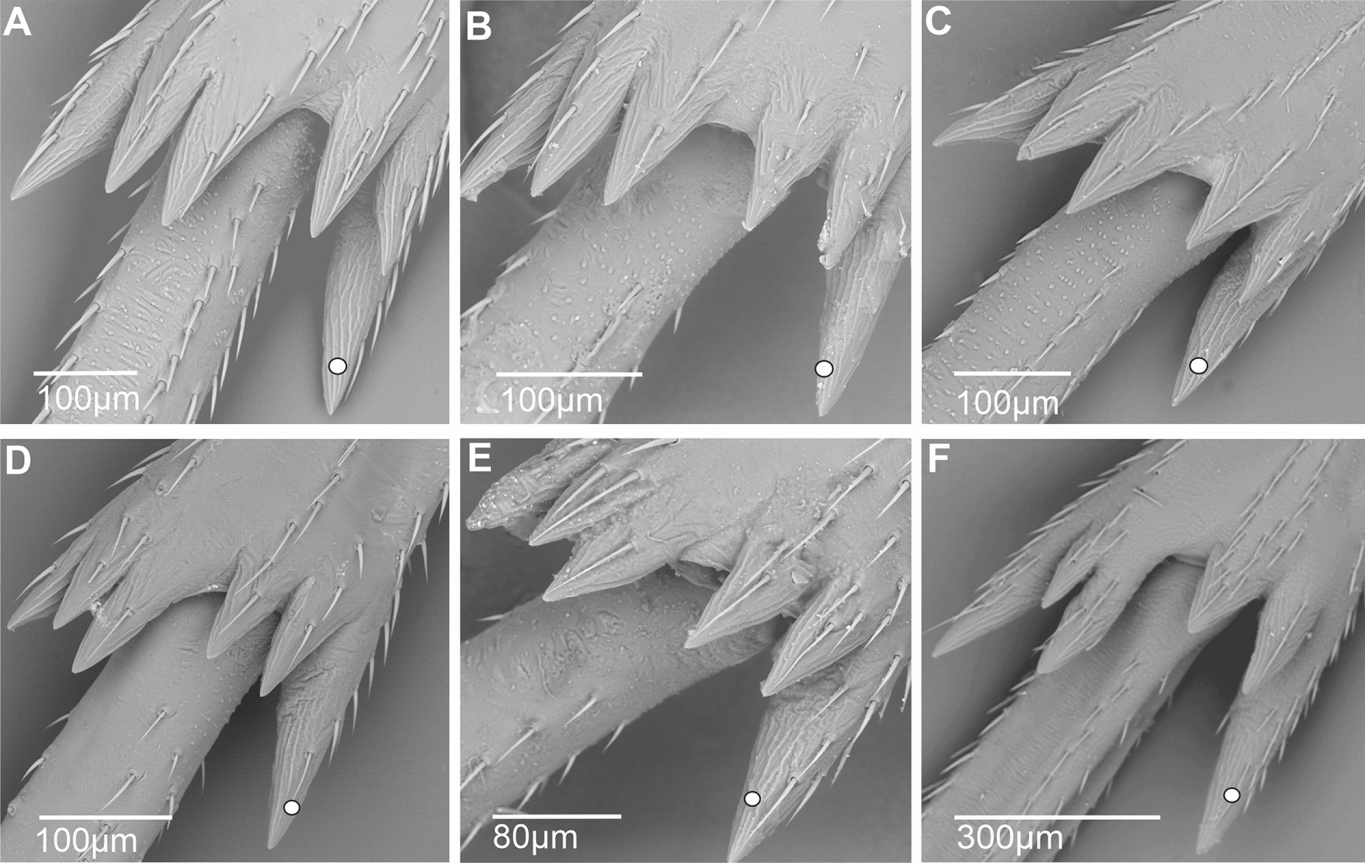
Distribution and types of the apical teeth on the tibia (ventral view). White dot indicates outermost external tooth. **A*** Pentastira rorida*. **B*** Oecleopsis artemisiae*. **C*** Reptalus panzeri*. **D*** Cixius pini*. **E*** Tachycixius pilosus*. **F*** Mnemosyne arenae*

A narrow diastema is sometimes due to a deeper incision observable in most Pentastirini: *Pentastira rorida* (Fig. 14A), *Oecleopsis artemisiae* (Fig. 14B), *Reptalus panzeri* (Fig. 14C), *R. quadricinctus*, and also some Cixiini such as *C. nervosus*,* C. pini* (Fig. 14D), *Tachycixius pilosus* (Fig. 14E), and in Mnemosynini *Mnemosyne arenae* (Fig. 14F).

**Fig. 15:**
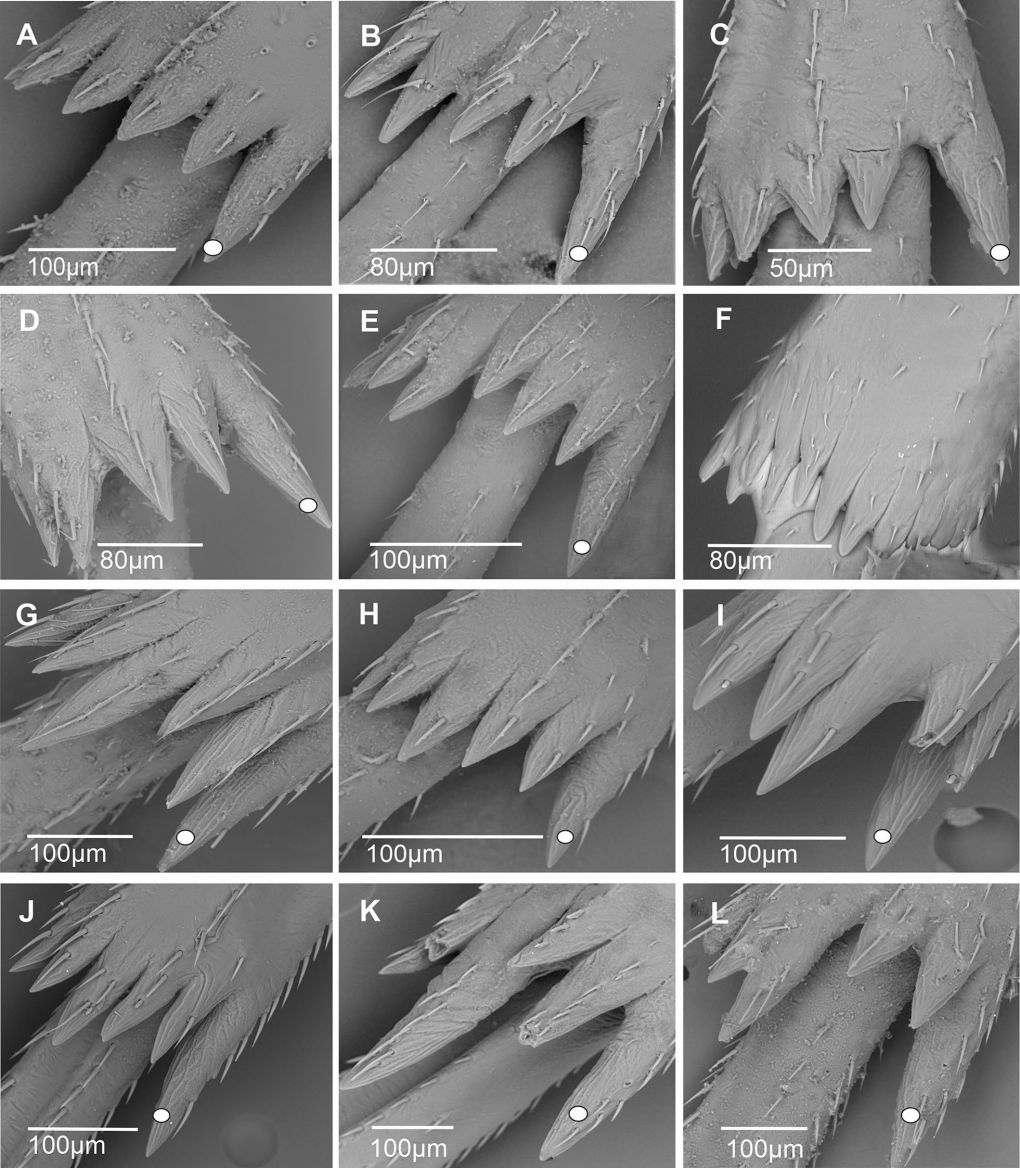
Distribution and types of the apical teeth on the tibia (ventral view). White dot indicates outermost external tooth. **A*** Borysthenes maculatus*. **B*** Duilius* (*Duilius*) *tatianae*. **C*** Leptolamia radicula*. **D*** Muirolonia metallica*. **E*** Duilius* (*Bitropis*) *fasciatus*. **F*** Euryphlepsia vangoethemi*. **G*** Brixidia variabilis*. **H*** Brixia* sp. **I**. * Andes marmoratus*. **J*** Noabennarella* sp. **K*** Benna* sp. **L*** Betacixius ocellatus*

In most Cixiidae taxa, the outermost tooth of the external group is the longer one of the six teeth as in Borysthenini (*Borysthenes lacteum*, *B. maculatus* (Fig. 15A). In Mnemosynini, the second tooth of the internal group is notably shorter.

The number of teeth can be reduced independently in various tribes: for instance, five spines were observed in Duiliini *Duilius* (*Duilius*) *tatianae* (Fig. 15B), Cixiini *Leptolamia radicula* (Fig. 15C), or Pintalini *Muirolonia metallica* (Fig. 15D). In these cases, the missing tooth belongs to the internal group of teeth. Conversely, the number of teeth may also increase, as in Duiliini *Duilius* (*Bitropis*) *fasciatus* (Fig. 15E), and an additional tooth belonging to the external group is observed. In Stenophlepsiini, an apomorphic condition is observed with the presence of 11 apical teeth of equal length, as in *Euryphepsia vangoethemi* (Fig. 15F). In Acrotiarini Bourgoin & Luo, 2021, the fossil genera *Pentacarinus*,* Acrotiara*, and *Maculixiu*s exhibit a typical condition with six apical teeth of equal length, with first latero-external one longer in *Acrotiara*, but with eight teeth observable in the genus *Delphitiara* [11].

**Fig. 16:**
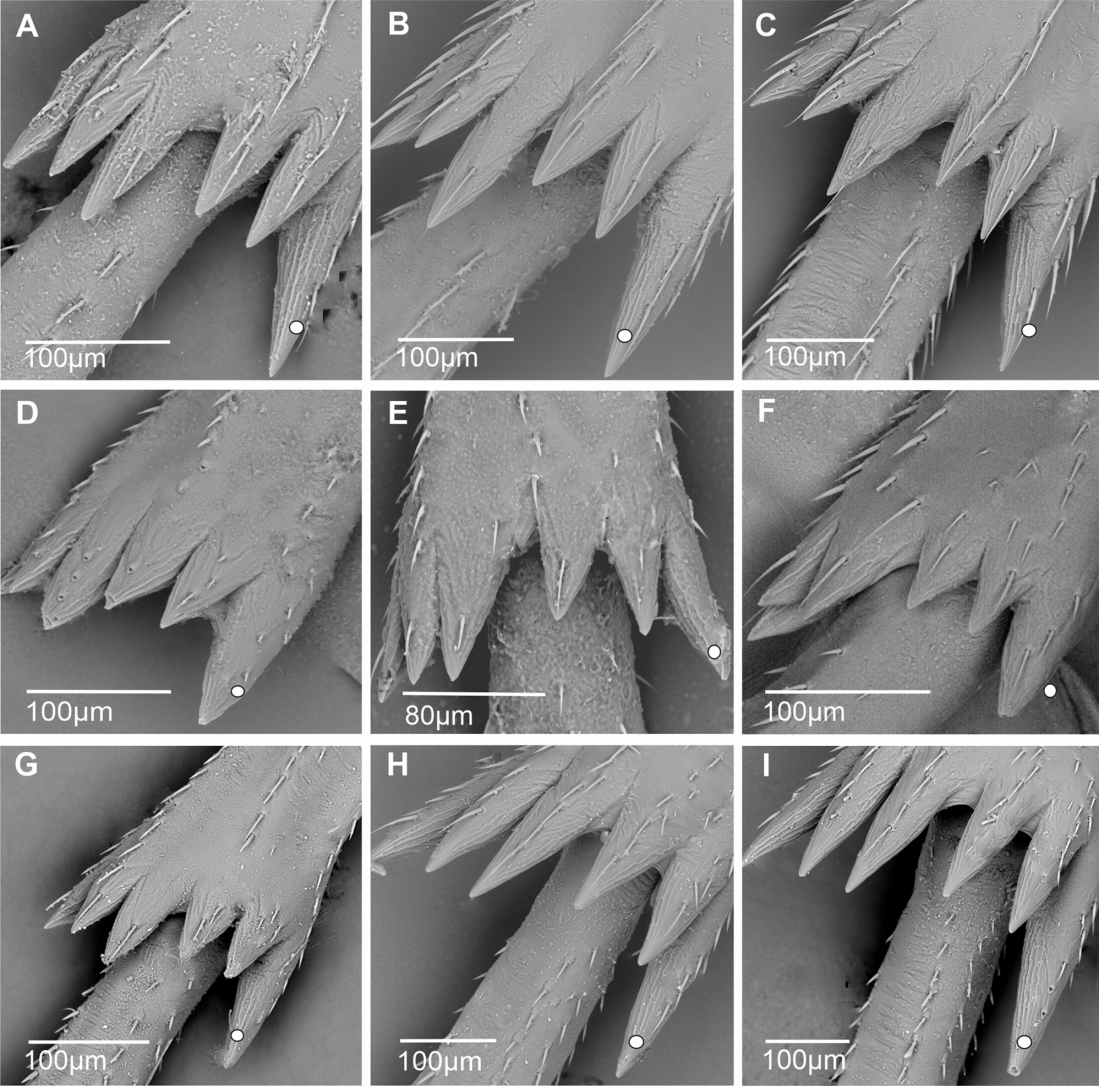
Distribution and types of the apical teeth on the tibia (ventral view). White dot indicates outermost external tooth. **A*** Kuvera tappanella*. **B*** Pintalia vibex*. **C*** Monorachis sordulentus*. **D*** Cubana* sp. **E*** Wernindia lorda*. **F*** Achaemenes kalongensis*. **G*** Macrocixius giganteus*. **H*** Oliarus annandalei*. **I*** Melanoliarus kindli*

In the internal group of teeth, the latero-external tooth is also often the longer one of the group. Such conformation is present in numerous taxa such as in Brixidiini *Brixidia boukokoensis* or *B. variabili*s (Fig. 15G), in Brixiini (*Brixia* sp., Fig. 15H), Andini (*Andes marmoratus*, Fig. 15I), Bennarellini (*Noabennarella* sp., Fig. 15J), or Bennini (*Benna* sp., Fig. 15K). A similar pattern is also observed in the Semonini *Betacixius ocellatus*, (Fig. 15L) or *Kuvera tappanella* (Fig. 16A), in the Pintaliini such as *Pintalia vibex* (Fig. 16B) *Monorachis sordulentus* (Fig. 16C) and *Cubana* sp. (Fig. 16D), in the Gelastocephalini *Wernindia lorda* (Fig. 16E), in *Achaemenes quienquespinus* and *A. kalongensis* (Fig. 16F), in the Cixiini *Cixiosoma bonaerense* or *Macrocixius giganteus* (Fig. 16G), and in the Pentastirini species such as *Oliarus annandalei* (Fig. 16H), *Hyalesthes lutipes*, *Pentastiridius beieri*, *Melanoliarus kindli* (Fig. 16I) and *M. complectus*.

**Fig. 17:**
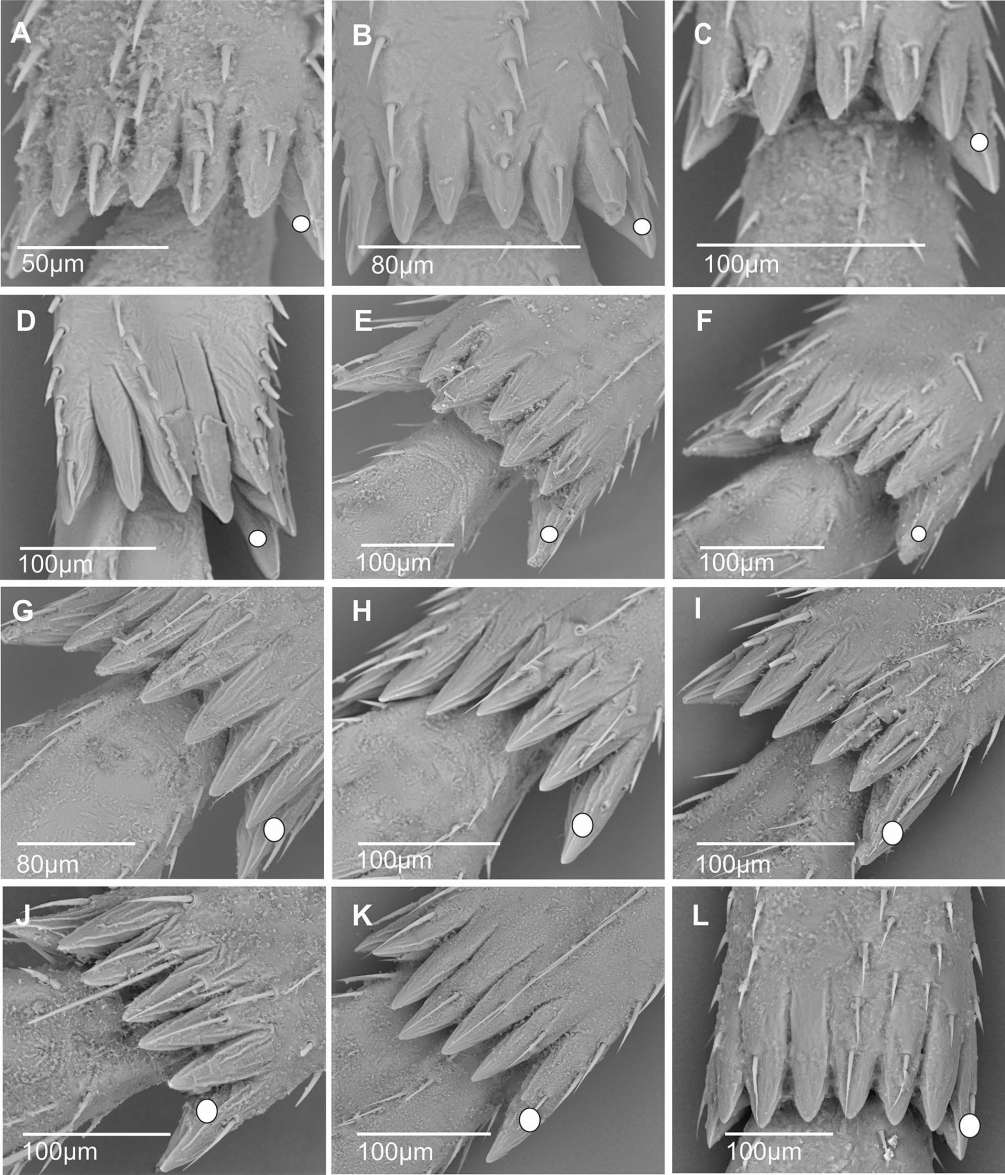
Distribution and types of the teeth on the first metatarsomere (ventral view). White dot indicates outermost external tooth. **A*** Borysthenes maculatus*. **B*** Bothriocera* sp. **C*** Brixia* sp. **D*** Benna* sp. **E*** Andes marmoratus*. **F*** Macrocixius giganteus*. **G*** Cixius nervosus*. **H*** Cixiosoma bonaerense*. **I*** Tachycixius pilosus*. **J*** Kuvera tappanella*. **K*** Pintalia vibex*. **L*** Notocixius helvolus*

**Fig. 18:**
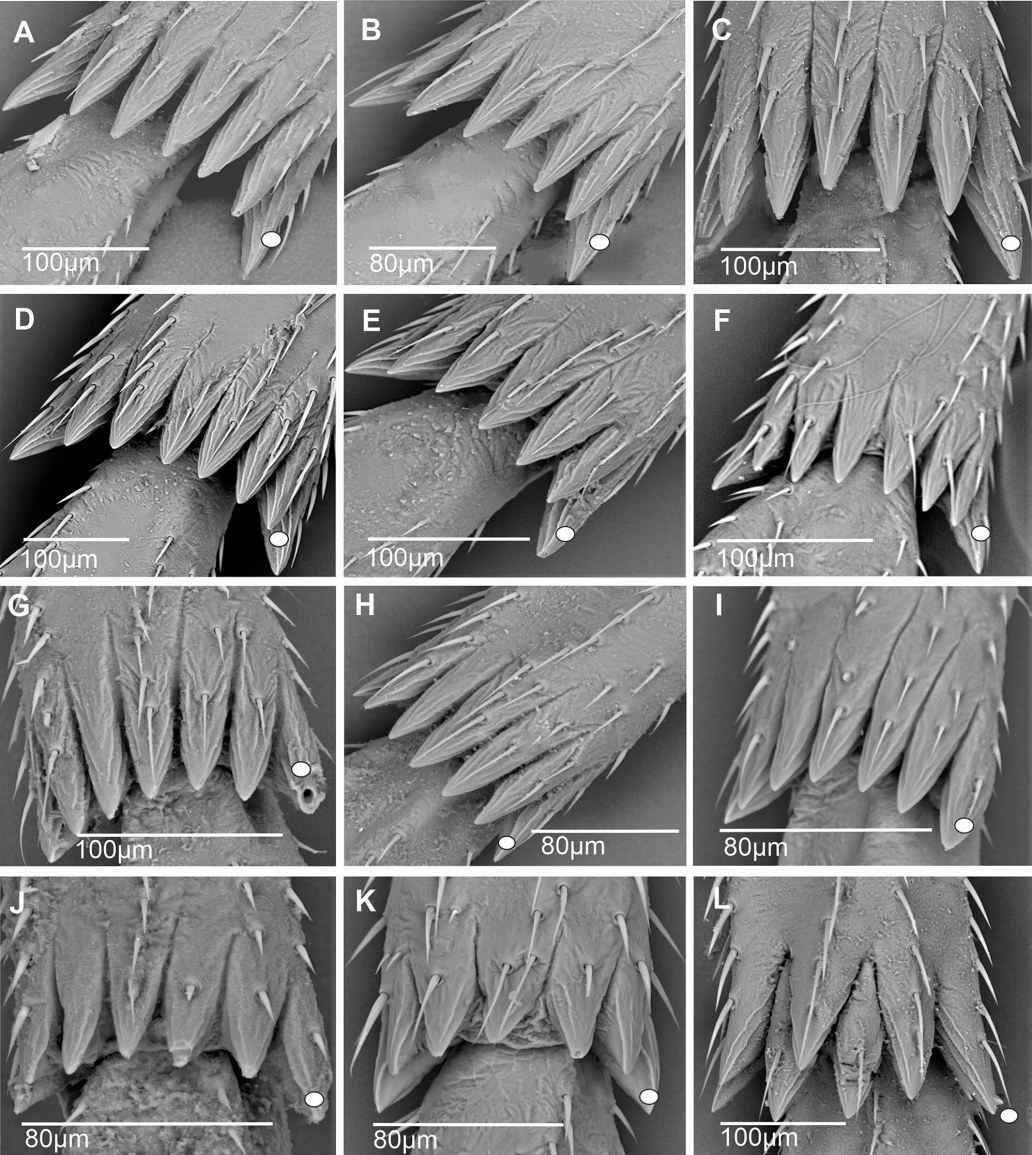
Distribution and types of the teeth on the first metatarsomere (ventral view). White dot indicates outermost external tooth. **A*** Melanoliarus kindli*. **B*** Oecleopsis artemisiae*. **C*** Oliarus annandalei*. **D*** Pentastira rorida*. **E*** Reptalus panzeri*. **F*** Setapius* sp. **G*** Haplaxius pictifrons*. **H*** Myndus musivus*. **I*** Nymphomyndus cribbea*. **J*** Mundopa kotoshonis*. **K*** Oecleus borealis*. **L*** Brixidia boukokoensis*

**Fig. 19:**
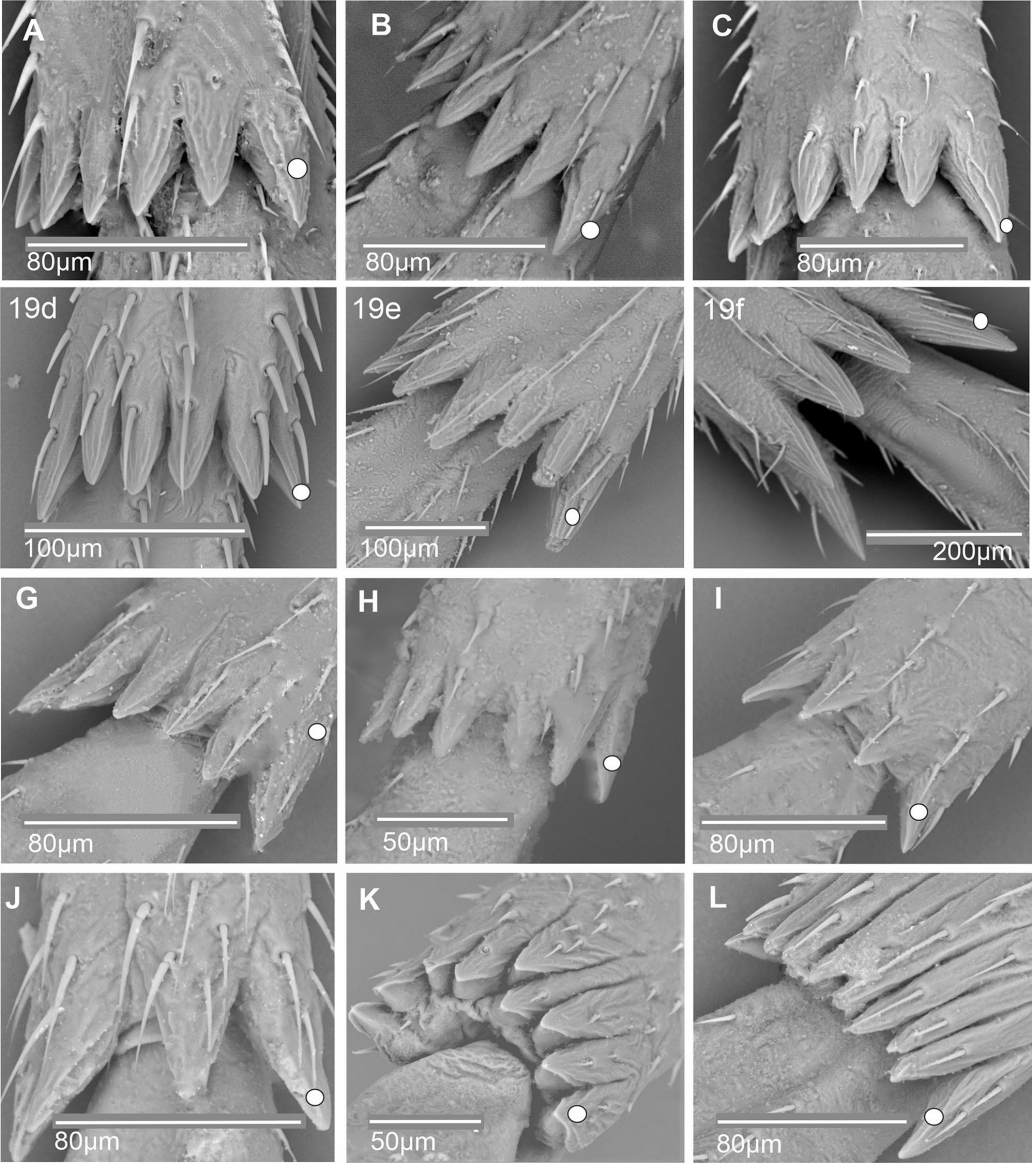
Distribution and types of the teeth on the first metatarsomere (ventral view). White dot indicates outermost external tooth. **A*** Achaemenes quienquespinos*. **B*** Achaemenes kalongensis*. **C*** Leptolamia radicula*. **D*** Noabenarella sp*. **E*** Betacixius ocellatus*. **F*** Mnemosynae arenae*. **G*** Duilius (Bitropis) fasciatus*. **H*** Coframalaxius bletteryi*. **I*** Trigonocranus emmae*. **J*** Duilius (Duilius) tatianae*. **K*** Myndus taffini*. **L*** Pinacites calvipennis*

**Fig. 20:**
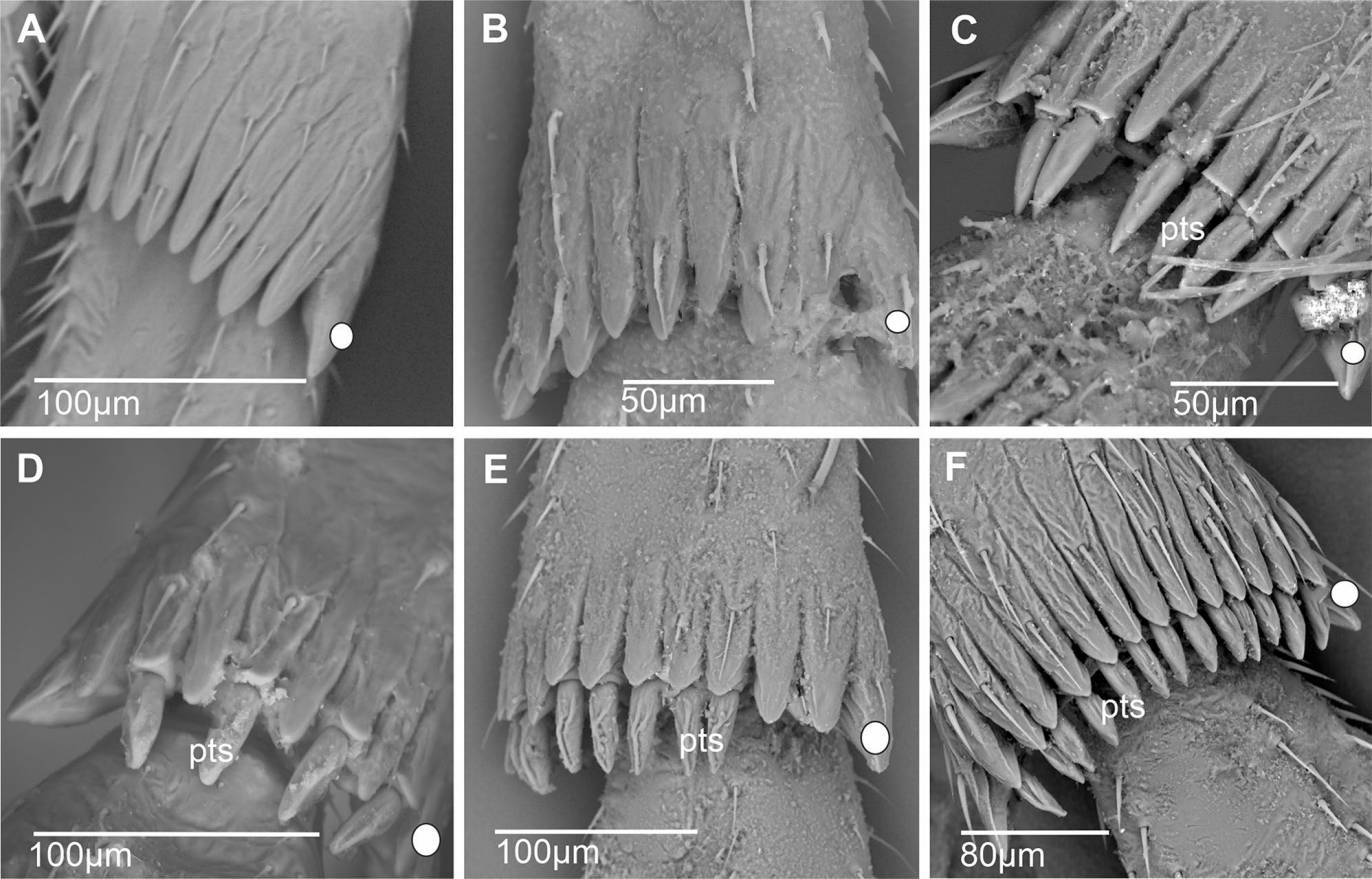
First metatarsomere posterior margin, ventral side: distribution of apical teeth and subdorsal platellar sensilla (ventral view). White dot indicates outermost external tooth. **A*** Euryphepsia vangoethemi*. **B*** Wermindia lorda*. **C*** Eucarpia elisabethana*. **D*** Gelastocaledonia monteithi*. **E*** Chidaea* sp. **F*** Pentastiridius leporinus*

**First metatarsomere.** The first metatarsomere in Cixiidae (Figs. 17, 18, 19 and 20) exhibits a relatively stable conformation and mainly diversifies by the number of their apical cuticular teeth (Table 3). When subdorsal sensilla are present, they never occur on the outer- and innermost lateral teeth.

**Table 3:** Number of teeth and presence of subdorsal sensilla (always of platellar type) observed by SEM analyses on the first metatarsomere in some cixiid taxa

Tribes or clades	Species (Figs nb)	Number of teeth	Subdorsal sensilla (distribution on teeth)
Duiliini	*Duilius* (*Duilius*) *tatianae* (Fig. 19J)	4	—
	*Duilius* (*Bitropis*) *fasciatus* (Fig. 19G)	6	—
Stenophlepsiini	*Euryphepsia vangoethemi* (Fig. 20A)	12	—
Oecleini	*Haplaxius pictifrons* (Fig. 18G)	7	—
	*Myndus musivus* (Fig. 18H)	7	—
	*Myndus taffini* (Fig. 19K)	9	—
	*Nymphocixia unipunctata*	7	—
	*Nymphomyndus caribbea* (Fig. 18I)	7	**—**
	*Pinacites calvipennis* (Fig. 19L)	10	—
	*Trigonocranus emmeae* (Fig. 19I)	4	—
	*Coframalaxius bletteryi* (Fig. 19H)	6	—
	*Meenocixius virescens*	7	—
	*Mundopa kotoshonis* (Fig. 18J)	7	—
	*Oecleus borealis* (Fig. 18K)	7	—
Bothriocerini	*Bothriocera* sp. (Fig. 17B)	8	—
Mnemosynini	*Mnemosyne arenae* (Fig. 19F)	5	—
Borysthenini	*Borysthenes maculatus* (Fig. 17A)	8	—
	*Borysthenes lacteus*	6	—
Pentastirini	*Pentastira rorida* (Fig. 18D)	8	**—**
	*Oecleopsis artemisiae* (Fig. 18B)	8	—
	*Hyalesthes luteipes*	7	**—**
	*Setapius* sp. (Fig. 18F)	8	**—**
	*Reptalus panzeri* (Fig. 18E)	7	**—**
	*Reptalus quadricinctus*	7	**—**
	*Oliarus annandalei* (Fig. 18C)	7	—
	*Pentastiridius beieri*	12	**10** (2–11)
	*Pentastiridius leporinus* (Fig. 20F)	15	**13** (2–12)
	*Melanoliarus kindli* (Fig. 18A)	7	—
	*Melanoliarus complexus*	9	—
	*Melanoliarus placitus*	6	—
Achaemenes + clade	*Achaemenes kalongensis* (Fig. 19B)	5	**—**
	*Achaemenes quinquespinosus* (Fig. 19A)	7	—
Chidaea + clade	*Chidaea* sp. (Fig. 20E)	10	**6** (4–9)
Pintaliini	*Pintalia vibex* (Fig. 17K)	7	—
	*Monorachis sordulentus*	7	**—**
	*Muirolonia metallica*	7	—
	*Notocixius helvolus* (Fig. 17L)	8	—
	*Cubana* sp.	8	—
Andini	*Andes marmoratus* (Fig. 17E)	7	—
Brixiini	*Brixia* sp. (Fig. 17C)	7	—
Gelastocephalini	*Gelastocaledonia monteithi* (Fig. 20D)	11	**4** (3,5,7,9)
	*Wernindia lorda* (Fig. 20B)	8	**—**
Eucarpiini	*Eucarpia elisabethana* (Fig. 20C)	11	**6** (3–6,8,9)
Bennini	*Benna* sp. (Fig. 17D)	8	—
Cixiini	*Cixius pini*	7	**—**
	*Cixius nervosus* (Fig. 17G)	8	**—**
	*Tachycixius pilosus* (Fig. 17I)	7	**—**
	*Macrocixius giganteus* (Fig. 17F)	8	—
	*Leptolamia radicula* (Fig. 19C)	6	**—**
	*Cixiosoma bonaerense* (Fig. 17H)	7	
Bennarellini	*Noabenarella* sp. (Fig. 19D)	6	—
Brixidiini	*Brixidia boukokoensis* (Fig. 18L)	7	—
	*Brixidia variabilis*	7	—
Semonini	*Betacixius ocellatus* (Fig. 19E)	6	—
	*Kuvera tappanella* (Fig. 17J)	7	

In most taxa, 7–8 apical metatarsomere teeth are observable, arranged in a single row, and without special subdorsal sensilla, although typical chaetic sensilla are usually present ventrally. This typical pattern is present in Borysthenini (*Borysthenes maculatus*, Fig. 17A), in Bothriocerini (*Bothriocera* sp., Fig. 17B), in Brixiini (*Brixia* sp., Fig. 17C), in Bennini (*Benna* sp., Fig. 17D), in Andini (*Andes marmoratus*, Fig. 17E), in Cixiini (*Macrocixius giganteus*, Fig. 13F), *Cixius nervosus* (Fig. 17G), and *C. pini*, *Cixiosoma bonaerense* (Fig. 17H), *Tachycixius pilosus* (Fig. 17I), in Semonini (*Kuvera tappanella*, Fig. 17J), in Pintalini (*Pintalia vibex*, Fig. 17K), *Notocixius helvolus* (Fig. 17L), *Monorachis sordulentus*, *Muirolonia metallica* and *Cubana* sp., in Pentastirini (*Melanoliarus kindli*, Fig. 18A) and *M. placitus*, *Hyalesthes luticeps*,* Oecleopsis artemisiae* (Fig. 18B), *Oliarus annandalei* (Fig. 18C), *Pentastira rorida* (Fig. 18D), *Reptalus panzeri* (Fig. 18E), *Setapius* sp., (Fig. 18F), in Oecleini: *Haplaxius pictifrons*, (Fig. 18G), *Myndus musivus* (Fig. 18H), *Nympomyndus cribbea* (Fig. 18I), *Mundopa kotoshonis* (Fig. 18J) and *Oecleus borealis* (Fig. 18K). From this main schema, a very peculiar apomorphic conformation is reported for *Brixidia boukokoensis* (Fig. 18L) for which the 7–8 apical teeth are arranged in two rows.

A pattern with a lower number of teeth from 5 to 6, in one row and without dorsal sensilla, has been observed in a few taxa: *Achaemenes quienquespinosus* (Fig. 19A) and *A. kalongensis* (Fig. 19B), in the Cixiini *Leptolamia radicula* (Fig. 19C), in Benarellini *Noabennarella* sp., (Fig. 19D), Semonini *Betacixius ocellatus* (Fig. 19E), Mnemosynini *Mnemosynae arenae* (Fig. 19F) and Duiliini *Duilius* (*Bitropis*) *fasciatus* (Fig. 19G). In the subtroglophile taxa five teeth (*Coframalaxius bletteryi*, Fig. 19H) or even four teeth only in *Trigonocranus emmeae* (Fig. 19I) were noticiable as well as in *Duilius* (*Duilius*) *tatianae* (Fig. 19J).

Patterns with a higher number of teeth are also reported, with 9–11 teeth in a single row and without dorsal sensilla in Oecleini *Myndus taffini* (Fig. 19K), *Pinacites calvipennis* (Fig. 19L), Stenophlepsiini *Euryphlepsia vangoethemi* (Fig. 20A) and Gelastocephalini *Wernindia lorda* (Fig. 20B).

In a few taxa with 10–11 apical teeth in a single row, subdorsal platellar sensilla are present on the first metatarsomere with different distributions according to the taxa. For instance, in Eucarpiini *Eucarpia elisabethana* (Fig. 20C) platellar sensilla are observed on teeth 3, 4, and 6, 7, 8, 9; in Gelastocephalini *Gelastocaledonia monteithi* (Fig. 20D) on teeth 3, 5, 7, 9; and in the Australian Cixiini group with *Chidaea* sp., (Fig. 20E) on teeth 4–8 and 10. In the Pentastrini *Pentastiridius leporinus* (Fig. 20F), a still more elaborate pattern in found with 15–16 apical teeth in one row, with 13 subapical dorsal patellar sensilla (pts2) except on the two lateral teeth.

**Fig. 21:**
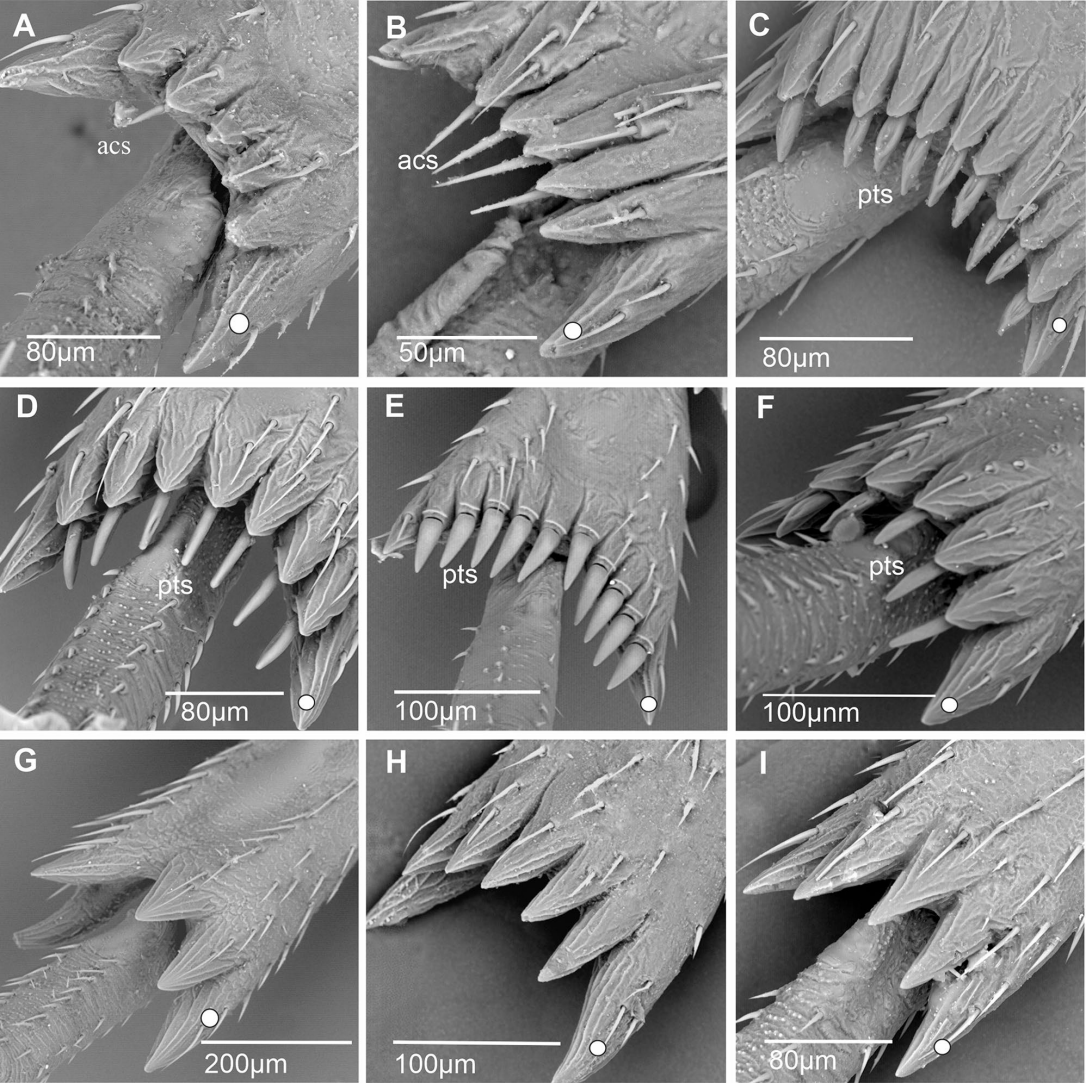
Shape of the apical teeth, acutellar sensilla (acs) and platellar sensilla (pts) on the second metatarsus. White dot indicates outermost external tooth. **A*** Andes marmoratus*. **B*** Pinacites calvipennis*. **C*** Pentastiridius leporinus*. **D*** Pentastira rorida*. **E*** Setapius* sp. **F*** Cixius nervosus*. **G*** Mnemosyne arenae*. **H*** Melanoliarus kindli*. **I*** Oecleopsis artemisiae*

**Second metatarsomere**. The diversity of Cixiidae is particularly well expressed in the conformation of the second metatarsomere. The shape of the apical teeth is generally roughly triangular elongated, which may be short, almost as long as wide (± 40 μm) such as in the Andini species *Andes marmoratus* (Fig. 21A) or more than four time longer than wide (± 80 μm) in Oecleini (*Pinacites calvipennis*, Fig. 21B) or in Pentastirini (*Pentastridius leporinus* (Fig. 21C), *Pentastira rorida* (Fig. 21D), *Setapius* sp., (Fig. 21E) and in the Cixiini species *Cixius nervosus* (Fig. 21F). The number of the apical teeth varies from five (in the Mnemosynini species *Mnemosyne arenae*, Fig. 21G) to 12 in several Cixiini and Pentastirini taxa of this study; however, the main pattern varies between seven to eight teeth.

Subdorsal acutellar sensilla are generally present but several taxa exhibit platellar sensilla; no chaetic sensilla are observed on the second metatarsomere. For one taxon, only one type of sensillum is observed: either acutellar sensillum (acs), either platellar sensillum (pts) (Fig. 7). Subdorsal sensilla are however absent in Mnemosynini *Mnemosyne arenae* Fig. 21G) and in some Pentastirini taxa, such as in *Melanoliarus kindli* (Fig. 21H), *M. placitus*, *Oliarus annandalei* and *Oecleopsis artemisiae* (Fig. 21I). As for the basimetatarsomere, subdorsal sensilla never occurs on the outer and innermost lateral teeth, except in *Coframalaxius bletteryi*, where the four teeth bear it, even on tooth 1.

Acutellar or platellar sensilla may be present in any teeth except in the innermost and outermost teeth. However, the great diversity of observed patterns prevents their classification into formal groups; Table 4 summarizes the encountered distributions.

**Table 4:** Number of teeth and presence of subdorsal sensilla, acutellar or platellar type, observed by SEM analyses on the first metatarsomere in some cixiid taxa

Tribes or clades	Species (Figs nb)	Nr. of teeth	Acutellar sensilla (distribution on teeth)	Platellar sensilla (distribution on teeth)
Duiliini	*Duilius* (*Duilius*) *tatianae*	5	**3** (2,3,4)	—
	*Duilius* (*Bitropis) fasciatus*	6	**4** (2,3,4,5)	—
Stenophlepsiini	*Euryphepsia vangoethemi*	8	**3** (3,4,6)	—
Oecleini	*Haplaxius pictifrons*	8	**3** (3,4,6)	—
	*Myndus musivus*	7	**3** (3,4,6)	—
	*Myndus taffini*	6	**2** (3,4)	—
	*Nymphocixia unipunctata*	7	**3** (3,4,6)	—
	*Nymphomyndus caribbea*	7	**3** (3,4,5)	**—**
	*Pinacites calvipennis* (Fig. 21B)	7	**3** (3,4,6)	—
	*Trigonocranus emmeae*	6	**2** (3,4)	—
	*Coframalaxius bletteryi*	4	**4** (1,2,3,4)	—
	*Meenocixius virescens*	7	**3** (3,4,5)	—
	*Mundopa kotoshonis*	8	**3** (3,4,6)	—
	*Oecleus borealis*	8	**6** (2–7)	—
Bothriocerini	*Bothriocera* sp.	7	**3** (3,4,6)	—
Mnemosynini	*Mnemosyne arenae* (Fig. 21G)	5	—	—
Borysthenini	*Borysthenes maculatus*	5–6	**1** (3)	—
	*Borysthenes lacteus*	5	present	—
Pentastirini	*Pentastira rorida* (Fig. 21D)	9	—	**7** (2–8)
	*Oecleopsis artemisiae* (Fig. 21I)	7	—	—
	*Hyalesthes luteipes*	8	—	**5** (3–7)
	*Setapius sp.* (Fig. 21E)	12	—	**10** (2–11)
	*Reptalus panzeri*	8	—	**5** (2–3,5–7)
	*Reptalus quadricinctus*	7	—	**5** (2–6)
	*Oliarus annandalei*	7	—	—
	*Pentastiridius beieri*	12	—	**8** (3–11)
	*Pentastiridius leporinus* (Fig. 21C)	12	—	**10** (2–11)
	*Melanoliarus kindli* (Fig. 21H)	7	—	—
	*Melanoliarus complexus*	8	**3** (3,4,6)	—
	*Melanoliarus placitus*	7	—	—
Achaemenes+	*Achaemenes kalongensis*	7	—	**4** (3,4,5,6)
	*Achaemenes quinquespinosus*	7	—	present
Australian Cixiini	*Chidaea* sp.	10	—	**8** (2–9)
Pintaliini	*Pintalia vibex*	8	—	**3** (3,4,5)
	*Monorachis sordulentus*	8	—	**3** (3,4,5)
	*Muirolonia metallica*	8	—	**3** (3,4,5)
	*Notocixius helvolus*	8	4 (3,4,5,6)	—
	*Cubana* sp.	8	**3** (3,4,6)	—
Andini	*Andes marmoratus* (Fig. 21A)	7	**3** (3,4,5)	—
Brixiini	*Brixia* sp.	8	**3** (3,4,6)	—
Gelastocephalini	*Gelastocaledonia monteithi*	10	—	**8** (2–9)
	*Wernindia lorda*	10	—	**8** (2–9)
Eucarpiini	*Eucarpia elisabethana*	11	—	**6** (3–6,8,9)
Bennini	*Benna* sp.	9	**4 (**3,4,5,7)	—
Cixiini	*Cixius pini*	7	—	**3** (3,4,5)
	*Cixius nervosus* (Fig. 21F)	7	—	**5** (2–6)
	*Tachycixius pilosus*	8	—	**3** (3,4,6)
	*Macrocixius giganteus*	9	—	**4** (3,4,5,7)
	*Leptolamia radicula*	7	—	**3** (3,4,5,7)
	*Cixiosoma bonaerense*	9	—	**6** (3–8)
Bennarellini	*Noabenarella* sp.	5	**1** (3)	—
Brixidiini	*Brixidia boukokoensis*	8	**3** (3,4,6)	—
	*Brixidia variabilis*	8	**3** (3,4,6)	—
Semonini	*Betacixius ocellatus*	7	**3** (3,4,5)	—
	*Kuvera tappanella*	8	—	**3** (3,4,6)

## Discussion

### Diversity of metatibiotarsal patterns observed in Cixiidae: tribal distribution

According to our SEM studies and the available literature, our results show that metatibiotarsal morphological structures are diversely distributed among Cixiidae taxa. Both tribes and genera exhibit a significant diversity of patterns, and display high homoplasy. Only their interpretation with reference to a solid phylogenetic framework would make it possible to distinguish between parallel evolutions and independent reductions of these morphological structures.

In this respect, and including the fossil tribe of Acrotiarini, the Cixiidae are currently classified into 19 tribes. In Emeljanov’s 2002 phylogram, four main subgroups are recognized (Fig. 1), however, they have not been confirmed by other molecular morphological and phylogenetic analyses [9–12]. Summarizing our current knowledge allowed to propose a provisional phylogenetical topology recognizing, moreover and at least, two other distinct clades: the Achemenes + and the Chidaea^+^ ones [11, 12, 14]; (Fig. 1). It would thus be rash to draw any hasty conclusions given the current state of knowledge.

However, with exception of the paraphyletic Oecleini including the Bothriocerini and the polyphyletic Cixiini split in several non-related groups and including Semonini, most tribes seem fairly well established and therefore could exhibit significant patterns. Accordingly, we explored whether some correlations could be highlighted between the patterns observed and the grouping of tribes according to Emeljanov [6] and the evolutionary lineages of Luo et al. [11] and clades recovered in Bucher et al. [12]. For that, a brief diagnosis of the metatibiotarsal models of each tribe (including the fossil tribe Acrotiarini) is here proposed in order to select certain general character states, which were later optimized on the last phylogenetical topology available for the Cixiidae, as proposed here (Fig. 1), and used as a frame of reference for interpreting our observations.

**Acrotiarini** A Cretaceous tribe with four fossil genera, tentatively placed at the base of the pentastirinian lineage by Luo et al. [11].

*Metatibiotarsal diagnosis*. Metatibia spiniform sensilla absent. With 6–9 apical teeth. No diastema. First metatarsomere with 7–11 apical teeth. With subdorsal platellar sensilla or without subdorsal sensilla (*Maculixius*). Second metatarsomere: with 6–10 apical teeth, with acutellar sensilla or platellar sensilla (*Delphitiara*).

**Andini** A small tribe currently with three genera and 135 species, absent from the Nearctic and Neotropical realms [16]. The tribe is placed at the base of group 3 of Emeljanov typology [6] successively with Brixini, Bennini and Brixidiini, but as an independent clade sister to Brixini [12].

*Metatibiotarsal diagnosis*. Metatibia with small and medium sized spiniform sensilla present. With six apical teeth. No diastema. First metatarsomere with eight apical teeth, without dorsal sensilla. Second metatarsomere with 7–8 teeth, subdorsal acutellar sensilla present.

**Bennarellini** A small Neotropical tribe of currently four genera separated from South-East Asian and Australian Bennini by Emeljanov [27]. Not placed in Emeljanov’s [6] and Bucher et al. ‘s [12] topologies.

*Metatibiotarsal diagnosis*. Metatibia with spiniform sensilla. Six apical teeth. No diastema. First metatarsomere with six apical teeth, without dorsal sensilla. Second metatarsomere: with five apical teeth; acutellar sensilla present in *Noabenarella*, absent in *Loisirella* Holzinger, Holzinger & Egger, 2013 [28].

**Bennini** South-East Asian and Australian tribe of 27 genera and 126 species [16], recently revised by Hoch [29]. Emeljanov placed as sister to Brixidiini in his third group of tribes [6]. Bennini is regards as sister clade to ‘true’ Cixiini according to Bucher et al. [12].

*Metatibiotarsal diagnosis*. Metatibia with numerous (11) small-sized spiniform sensilla present. Six apical teeth. No diastema. First metatarsomere: with eight apical teeth, without subdorsal sensilla. Second metatarsomere: with nine teeth; acutellar sensilla present.

**Borysthenini** A monogeneric tribe of 25 species distributed in the Oriental and African realms [16] separated as a subfamily [27] and followed downgraded to the tribe [11]. The taxon was tentatively attributed to the pentastirinian lineage, and Bucher et al. [13] confirmed the taxa as sister to the Pentastirini.

*Metatibiotarsal diagnosis*. Metatibia with small-sized spiniform sensilla present and five apical teeth, without diastema. Emeljanov [27] mentions however the absence of lateral teeth and six (1 + 5) apical teeth on the metatibia. First metatarsomere with seven apical teeth, without dorsal sensilla. Second metatarsomere with five teeth and one acutellar sensillum on the median tooth.

**Bothriocerini** A very distinctive group of Neotropical genera, also known from Western Europe as Eocene fossil [30, 31]. It is separated since Muir [1], upgraded to subfamily by Metcalf [30] and downgraded again to tribe by Luo et al. [11]. According to data analysis [12, 13], Bothriocerini nests in the Oecleini tribe, making it paraphyletic, although they are regarded as sister tribes by Emeljanov [6]).

*Metatibiotarsal diagnosis* Metatibia with numerous (11) small-sized spiniform sensilla present (versus absent in Emeljanov, [27]). Six apical teeth, with a diastema bearing one acutellar sensillum. First metatarsomere with 7–8 apical teeth, without dorsal sensilla; second with 5–8 teeth and acutellar sensilla present, (or absent, Emeljanov, [27]).

**Brixidiini** A monogeneric tribe of 12 Afrotropical species, established as a sister tribe to Bennini [6]. Not tested in Bucher et al.‘s phylogeny [12].

*Metatibiotarsal diagnosis*. Numerous (11) metatibial small-sized spiniform sensilla present. Six apical teeth. No diastema. First metatarsomere: with seven apical teeth in two rows, without dorsal sensilla. Second metatarsomere: with eight teeth, acutellar sensilla present.

**Brixiini** The tribe currently groups nine genera and 158 species, 114 of them belonging to the genus *Brixia* Stål, 1856 [16]. The tribe is distributed in the Afrotropical, Oriental and the Australian region from which the fauna was revised [32]; Brixini were placed sister to (Bennini + Brixidiini) by Emeljanov [6] and sister to Andini [12].

*Metatibiotarsal diagnosis*. Metatibia with small-sized spiniform sensilla. Six apical teeth. No diastema. First and second tarsomere with 7–8 apical teeth, acutellar sensilla present on the second.

**Cajetini** The tribe is monospecific, represented by a single species, *Cajeta singularis* Stål, 1866. No SEM observation was done, and data reported here are from Löcker et al. [33]. The tribe is considered sister to the clade (Stenophlepsiini + (Oecleini + Bothriocerini) by Emeljanov [6], and was therefore tentatively placed in the oecleinian lineage by Luo et al. [11]. Not tested by Bucher et al. [12].

*Metatibiotarsal diagnosis*. Metatibia without spiniform sensilla, with more than eight apical teeth, no diastema. First metatarsomere with 12 apical teeth, without subdorsal sensilla. Second metatarsomere with 11–15 apical teeth, with more than nine subdorsal sensilla.

**Cixiini** The tribe is based on its type genus *Cixius* Latreille 1804, one of the older genus of the family in which still about 300 species are placed, strongly altering a clear taxonomic concept of the tribe. According to Bucher et al. [12], the tribe is polyphyletic including non-related groups such as Achaemenes + and Chidaea^+^ clades. Emeljanov [34] specified that in its current concept, the genus *Cixius* is absent from Australia, whose species belongs to the genus *Chidaea* Emeljanov, 2000, as confirmed by Löcker and Holzinger [35]. According to Bucher et al. [12] the Australian Cixiini or Chidaea^+^ clade (*Chidaea*,* Tyligma* Löcker & Holzinger, 2020, *Leades* Jacobi, 1928) separate independently from “true Cixiini” as sister to Pintaliini, in a basal position in the cixiinian lineage. The “true Cixiini” group appears itself paraphyletic, including Semonini tribe. Polyphyly of the tribe seems well confirmed by the wide diversity of the patterns observed, which cannot be summarized at this stage in different groups. Some examples are provided here with annotations:

***Cixius nervosus*(Linné**,** 1758)** Type species of the genus and tribe. Metatibia with three spiniform sensilla. Six apical teeth in two groups, separated by a narrow diastema. First metatarsomere with eight apical teeth (7 in *C. pini* Fitch, 1851), without subdorsal sensilla. Second metatarsomere with 12 teeth, nine platellar sensilla on teeth 3–11 in *C. nervosus*; seven teeth, three platellar sensilla on teeth 3 to 5 in *C. pini*.

***Achaemenes*Stål**,** 1866** The single genus of the clade groups some 45 species is distributed in the Afrotropical realm, including Madagascar [16]. It is currently classified within the Cixiini [6]. In phylogeny, it is placed at the base of the Cixiinian lineage, as a sister group to all other tribes of the lineage, and independently from the other Cixiini [12]. Metatibial spiniform sensilla absent. Six apical teeth in two groups, first latero-external one of each group longer. No diastema. First metatarsomere with eight apical teeth, without subdorsal sensilla. Second metatarsomere with 7–8 teeth, and platellar sensilla.

***Chidaea*Emeljanov**,** 2000** As for *Achaemenes*, the genus belongs to an independent clade grouping Australian Cixiini; it is depicted as a sister clade to Pintaliini [12]. The lineage exhibits two short-sized spiniform sensilla, present in middle part of the metatibia. Six apical teeth in two groups separated by a wide diastema. First and second metatarsomeres with 11 and 10 apical teeth respectively, both with subdorsal platellar sensilla.

***Cixiosoma* Berg**,** 1879** Metatibia with three spiniform sensilla. Six apical teeth in two groups without diastema. First metatarsomere with 7 apical teeth, without subdorsal sensilla.

Second metatarsomere with 9 teeth, and subdorsal platellar sensilla.

***Tachycixius* Wagner**,** 1939** Metatibia with three medium-sized spiniform sensilla and six apical teeth separated by a narrow diastema. First metatarsomere with 7 apical teeth, without subdorsal sensilla; second with 8 teeth, and subdorsal platellar sensilla.

***Macrocixius* Matsumura**,** 1914** Metatibia with four metatibial spiniform sensilla and six apical teeth without diastema. First metatarsomere with 8 apical teeth, without subdorsal sensilla. Second with 9 teeth, and subdorsal platellar sensilla.

***Leptolamia* Metcalf**,** 1936** the genus was previously separated from *Bajauana* in Eucarpini from which the genus should probably return. Metatibial spiniform sensilla absent. Five apical teeth without diastema. First metatarsomere with 7–8 apical teeth, without subdorsal sensilla. Second metatarsomere with 7 teeth and subdorsal acutellar sensilla.

**Duiliini** A monogeneric tribe distributed in the Palaearctic and Afrotropical realms recognized by Emeljanov [6]. The genus is divided in three subgenera, which probably should be better consider as separate genera. The placement of the tribe at the base of the oeclinian lineage remains in doubt; it was not included in Bucher et al. [12] phylogeny. Following Emeljanov (2002), it is supposed to be part of the Oecleinian lineage [11].

*Metatibiotarsal diagnosis* Metatibia with sensilla spiniformia absent in subgenus *Duilius*, with two in the subgenus *Bitropis* Dlabola, 1985. Five apical teeth in subgenus *Duilius*, 7 in *Bitropis*. No diastema. First metatarsomere with four apical teeth in subgenus *Duilius*, six in *Bitropis*. Both without dorsal sensilla. Second metatarsomere with six teeth and four acutellar sensilla in subgenus *Duilius*; with five teeth and three acutellar sensilla in *Bitropis*. Five teeth on both metatarsomeres in the type species of the genus *Duilius tenuis* Stål, 1858 [36].

**Eucarpiini** The tribe groups currently 13 genera and 160 species mainly distributed in the Oriental and Australian realms, but with a few taxa occurring in Western Africa [16]. Eucarpini belongs to the cixiinian lineage placed as sister to Pintaliini by Emeljanov [6], and to (Bennini + (Cixiini + Semonini)) by Bucher et al. [12]. The tribe might be paraphyletic as the African *Eucarpia* species may not be congeneric with the Oriental ones, and probably do not even belong to Eucarpiini. Bucher et al. [12] phylogeny was based on Chinese specimens while the SEM images presented here on an African one.

*Metatibiotarsal diagnosis* Metatibia without spiniform sensilla. Six apical teeth generally, five in *Nesochlamys* Kirkaldy, 1907 [37]. No diastema. First metatarsomere with 6–7 apical teeth, six in *Bajauana* Distant,1907 and *Nesochlamys*; seven in *Dilacreon* Fennah, 1980, *Neocarpia* Tsaur & Hsu, 2003; without subdorsal sensilla. Second metatarsomere with 6–7 apical teeth, with subdorsal acutellar sensilla [37]. First and second metatarsomere with 11 apical teeth in the African species *Eucarpia elisabethana* (Synave, 1962), and both with subdorsal platellar sensilla.

**Gelastocephalini** A diversified tribe of 27 genera and 60 species distributed in the Australian realm [16] containing two subtribes Gelastocephalina and Rhigedanina [34], the first found to be paraphyletic [38]. The tribe was not positioned in Emeljanov’s [6] topology, but found its place in the cixiinian lineage in Bucher et al. [12].

*Metatibiotarsal diagnosis*. One proximal metatibial spiniform sensillum. A narrow diastema separating six apical teeth. First metatarsomere with 9–11 apical teeth, with subdorsal platellar sensilla in *Gelastocaledonia* sp., but absent in *Wernindia lorda* Löcker & Fletcher, 2006 [33]. Second metatarsomere with 9–10 apical teeth, and subdorsal platellar sensilla.

**Mnemosynini** A small group of six genera, five of which being Eocene fossils of Western Europe. The type genus *Mnemosyne* groups 50 + species distributed in the Neotropical, Afrotropical and Oriental regions, and two more species were recently described from Australia [39]. The genus is in need of revision, grouping very probably several distinct genera [33, 40–42]. The taxon was separated from Pentastirini as a subtribe [43], later upgraded to tribe [41], and supposed belonging to the pentastirinian lineage [11]. It was however found closer to the oecleinian lineage [12], although the authors specified that the result needs to be confirmed as the tribe was sampled by only one species.

*Metatibiotarsal diagnosis* Two long-sized metatibial spiniform sensilla. Six apical teeth in two groups with first latero-external one of external group longer and second of internal group distinctly shorter than other teeth. A wide diastema generally present (narrow in the Australian species *M. alexandri* Löcker, 2006). First and second metatarsomeres with five apical teeth; without subdorsal sensilla.

**Oecleini** A paraphyletic taxon including Bothriocerini [12–14], which is treated separately in this paper.

*Metatibiotarsal diagnosis* Metatibia spiniform sensilla most often absent (*Haplaxius* Fowler, 1904, *Myndus*, *Nymphocixia* Van Duzee, 1923, *Nymphomyndus* Emeljanov, 2007, *Pinacites* Emeljanov, 1972, *Trigonocranus* Fieber, 1875, *Coframalaxius* Bourgoin & Le Cesne, 2022, *Meenocixius* Attié, Bourgoin & Bonfils 2002) but with two shortspiniform sensilla in *Mundopa* Distant 1906 and 5 short-sized in *Oecleus* Stål, 1862; also present in *Confuga*. With six apical teeth. Diastema present. First metatarsomere without subdorsal sensilla. 5–10 teeth according to the genera: *Meenocixius* (7–8), *Myndus taffini* Bonfils, 1983 (9); *Pinacites* (10); *Trigonocranus* (5); *Coframalaxius* (6–9 teeth according the specimen). Second metatarsomere: with 7–8 apical teeth, with acutellar sensilla (*Oecleus*,* Coframalaxius*).

**Pentastirini** A major cixiid tribe of 44 genera and more than 800 species [16]. Although the monophyly of the tribe seems well assured, several different patterns are observed. It is placed as a a sister of (Cixiini + Semonini) in the fourth group by Emeljanov [6], while classified it in its own lineage with Mnemosynini and Borysthenini in recent phylogenies [11, 12]. As for Cixiini the disparity of the patterns observed prevent to summarize them in subgroups at this stage.

*Metatibiotarsal diagnosis*. Metatibial spiniform sensilla present in various numbers: according to the genera: three in *Oliarus* Stål, 1962, *Pentastira*,* Reptalus*, and *Setapius*. Also, three in the genera *Manurevana* Hoch, 2006 and *Oetana* Hoch, 2006 but for some species from Moorea Island their number varies from one to three [44]. Four in *Melanoliarus* Fennah, 1945 and *Pentastiridius*; five in *Hyalesthes*; eight in *Oecleopsis* Emeljanov, 1971. Diastema absent in *Hyalesthes*, *Oliarus*, and *Pentastiridius*; narrow one in *Oecleopsis*,* Melanoliarus*,* Pentastira*, and *Reptalus*; *Setapius* with a wide diastema. Six apical metatibial teeth in two groups; first latero-external one of external group longer. First metatarsomere, without subdorsal sensilla. Seven apical teeth in *Hyalesthes*, *Melanoliarus*, *Oliarus*, and *Reptalus*; eight in *Oecleopsis*, *Pentastira*, and *Setapius*. Genus *Pentastiridius* with more than 15 teeth with platellar sensilla on all teeth except the two lateral ones. Second metatarsomere: five apical teeth without dorsal sensilla in *Oecleopsis* and *Oliarus*, as well as the Oriental genera *Siniarus* Emeljanov, 2007 and *Arosinus* Emeljanov, 2007. Seven apical teeth in *Atretus* Emeljanov, 2007 and *Hyalesthes* with four platellar sensilla on teeth 3, 4, 5, 6 ; also seven apical teeth in *Melanoliarus kindli* Bourgoin, Wilson & Couturier, 1998, but without dorsal sensilla, such as in *Oteana* and *Manurevana* [44]; *M. complectus* (Ball, 1902) with eight teeth and three acutellar sensilla on teeth 3, 5, 6. *Reptalus* with eight apical teeth with platellar sensilla on all teeth except the two lateral ones. *Pentastira* with nine teeth with seven subdorsal platellar sensilla on teeth 2–8. *Pentastiridius* and *Setapius* with 12 apical teeth with platellar sensilla on all teeth except the two lateral ones.

**Pintalini** The tribe currently groups eight genera and 113 species, distributed in the New World and is mainly neotropical [16], although one Eocene fossil genus, *Worodbera* Szwedo, 2019, was described from Western Europe [31]. In the cixiinian lineage, the tribe is separated as sister to Eucarpini in group 2 of Emeljanov’s [6] phylogeny (Fig. 1), but as sister to the ‘Australian Cixiini’ clade in [12].

*Metatibiotarsal diagnosis* Three small- or medium-sized metatibial spiniform sensilla (absent in *Muirolonia*). Six apical teeth and no diastema in genera *Cubana*,* Pintalia*, and *Monorachis*; *Notocixius* and *Muirolonia* with a diastema and five apical teeth. First metatarsomere without subdorsal sensilla and 7–9 apical teeth. Second metatarsomere with eight apical teeth, with acutellar or platellar sensilla.

**Semonini** A mostly Oriental tribe currently grouping five genera and 61 species [16], and separated by Emeljanov from the Cixiini on the base of a swollen clypeus, a convex clypeofrontal margin and an obscure boundary between frons and vertex [6]. The tribe was found to be paraphyletic [12] with the genus *Kuvera* Distant, 1906, moved into the Cixiini, and moreover rendering the ‘true’ Cixiini tribe paraphyletic.

*Metatibiotarsal diagnosis*. Four to six medium-sized metatibial spiniform sensilla present. Six apical teeth, without diastema. First metatarsomere with 6–8 apical teeth without subdorsal sensilla. Second metatarsomere with eight apical teeth; with acutellar sensilla (*Betacixius*) or platellar sensilla (*Kuvera*).

**Stenophlepsiini** A peculiar cixiid South-East Asian tribe grouping two very distinctive genera and 14 species. Following Emeljanov [6], the tribe was placed in the oecleinian lineage by Luo et al. [11], sister to (Oecleini + Bothriocerini). This placement was not tested in the molecular phylogeny of Bucher et al. [12]. The tribe can be diagnosed by its metatibiotarsal conformation with numerous apical teeth on the tibia and the first two metatarsomeres.

*Metatibiotarsal diagnosis*. Metatibia without spiniform sensilla, with 11 apical teeth; no diastema. First metatarsomere with 12 apical teeth, without subdorsal sensilla. Second metatarsomere with nine apical teeth, with three subdorsal acutellar sensilla.

### Interpretation of the patterns observed in the light of the current phylogeny of Cixiidae

**Fig. 22:**
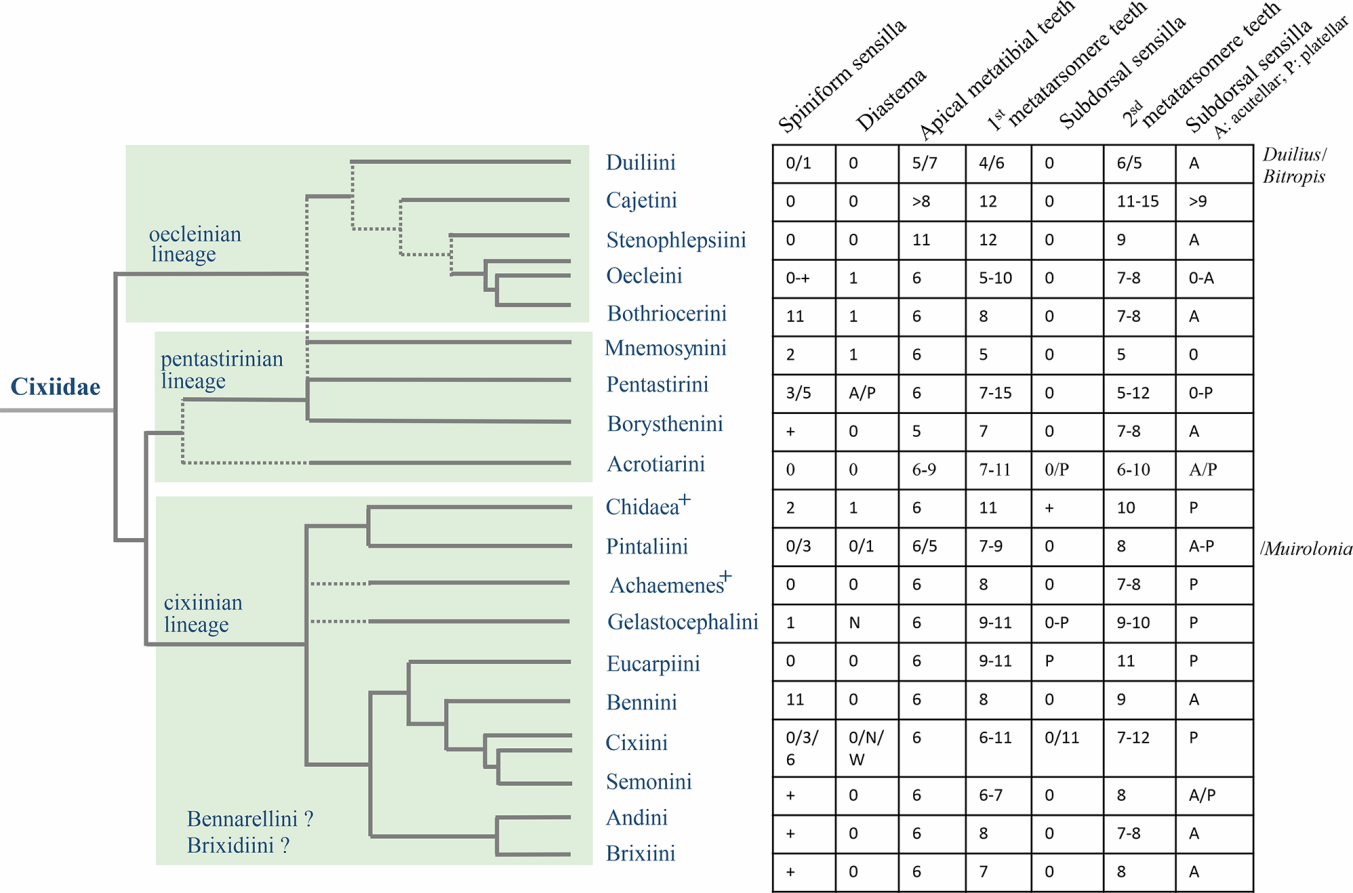
Diversity of metatibiotarsal patterns observed according to the tribe relationships in Cixiidae. The phylogeny is derived from the planthopper phylogeny presented by Bucher et al. [12], with slight modifications according to Emeljanov [6], Luo et al. [11], and Bourgoin et al. [14]. We only used published results on which we plotted K states. Abbreviations: A, absent; N, narrow; P, present; W, wide

In Cixiidae Emeljanov [25] first reported acutellar sensilla in the genera *Cixius* Latreille 1804, *Myndus* Stål, 1862 and *Eumecurus* Emeljanov, 1971, while platellar sensilla were found in *Pentastiridius* Kirschbaum, 1868, *Reptalus* Emeljanov, 1971, and *Hyalesthes* Signoret, 1865. Metatibial microcuticular ornamentations in planthoppers are in fact very diverse, both in the structures involved, cuticular expansions or sensory sensilla, but also in the patterns of their distributions, making it challenging to categorize all observed situations in distinct lineages. Nevertheless, we attempted to interpret our observations in light of the current phylogeny of Cixiidae, acknowledging the provisional nature of this topology, subject to modifications with more representative and diverse sampling in the future. Figure 22 illustrates the observed patterns by tribes on the phylogeny.

Spiniform sensilla were addressed by Emeljanov [5] mentioning that they first appear with the second instar in Cixiidae (on the third one in some delphacids). However, in 2002 he precised that these spines are unique for Cixiidae, representing a reversal autapomorphy for the family according to a reversal to the ancestral state as he schematized in his “revertive evolutionary (morphocyclic) modifications of armature of legs” scenario ([5], Fig. 17.1). Indeed, spiniform sensilla seem to occur only Cixiidae, and they were not observed in Delphacidae. However, they are not present in all cixiid clades, and one cannot state at this stage that they might represent an apomorphic character of the family, they might have evolved independently in all cixiid lineages.

The evaluation of the diastema proposed as plesiomorphic characters [6] remains challenging, as narrow gaps are frequently observed between the two groups of metatibial apical spines. A true diastema, as wide as at least the basal width of a tooth, is present in the related Oecleini and Bothriocerini. However, it also occurs independently in Mnemosynini and in the Australian group of Cixiini. In the tribe Pintaliini, the genus *Muirolonia* distinctly separates from other genera by having such a diastema.

The number of apical metatibial teeth appears to be relatively stable in the family, with a likely plesiomorphic condition of six teeth divided into two groups of three teeth each. The higher number of teeth observed in Stenophlepsiini and Cajetini is distinctive, but similar patterns are quite common in fossil planthoppers, such as in mid-Cretaceous Cixiidae Acrotiarini [11], suggesting that the 6-teethed metatibia might be a derived character. The presence of chaetic sensilla and sarcosetae on the dorsal side of apical metatibial teeth in extinct Cretaceous families and their absence in the family Cixiidae, which is basal to extant families, requires further research.

The number and conformation of teeth on the first and second metatarsomere vary significantly according to the tribe and even within genera. While these characters have proven useful for species or even genus identification, they may result from too many homoplasies for deeper phylogenetic analyses. However, the presence/absence of a subdorsal sensilla on the metatibial teeth, such as in some Lalacidae or in Cixiidae on the first metatarsomere (present only in the fossil tribe Acrotiarini and in Gelastocephalini and Eucarpiini), might carry some phylogenetic value that remains to be tested with larger phylogenetic analyses. In contrast, occurrence of these subdorsal as platellar or acutellar sensilla occurring on the second metatarsomere in nearly all tribes of Cixiidae makes their interpretation more challenging.

## Conclusions

It is interesting to note that the great diversity of sensory and non-sensory cuticular metatibiotarsal structures observed in cixiids does not allow for the emergence of clear evolutionary trends. This observation may be related to Emeljanov’s opinion [5], which highlighted multiple convergences and evolutionary regressions behind the observed patterns. He showed that the disparity and diversity of the metatibiotarsal structures, also observed in other planthopper families, increase with the development stage. He suggested an ontogenetic development of them in the planthopper family Dictyopharidae Spinola, 1839 [5], (Figs. 17 and 18). Among the Cixiidae, these highly diverse observations may also be linked to their way of life, as their larvae typically feed on underground roots. Indeed, most of them exhibit tendencies toward underground life with more or less pronounced troglomorphies (faintly colored integument, absence or very few compound eye ommatidia until the third stage, etc.), probably due to heterochronic effects of development [45]. These factors might have also influenced the expression of genes governing the diversity of the metatibiotarsal structures in Cixiidae.

Given our knowledge of sufficiently precise observations of the metatibiotarsal structures in the Cixiidae on the one hand, and the provisional phylogeny of the group as a frame of reference for their interpretations on the other hand, we can only conclude that the observed patterns are the result of multiple and independent evolutionary convergences and regressions. Although these models may be useful for the identification of taxa at a low taxonomic level, they may be less suitable for phylogenetic purposes.

## Data Availability

All data obtained and analysed during this study are available in this article.
